# Using high frequency GPS data to assess wintering goose proximity to commercial poultry facilities on the Delmarva peninsula for avian influenza risk management and surveillance

**DOI:** 10.1371/journal.pone.0355415

**Published:** 2026-09-01

**Authors:** Matthew J. Hardy, Christopher K. Williams, Brian S. Ladman, Maurice E. Pitesky, Cory T. Overton, Michael L. Casazza, Elliott L. Matchett, Diann J. Prosser, Pierre Legagneux, Josée Lefebvre, Jeffrey J. Buler

**Affiliations:** 1 Department of Entomology & Wildlife Ecology, University of Delaware, Newark, Delaware, United States of America; 2 Department of Animal & Food Sciences, University of Delaware, Newark, Delaware, United States of America; 3 University of California – Davis, School of Veterinary Medicine, Davis, California, United States of America; 4 U.S. Geological Survey, Western Ecological Research Center, Dixon Field Station, Dixon, California, United States of America; 5 U.S. Geological Survey, Eastern Ecological Science Center at the Patuxent Research Refuge, Laurel, Maryland, United States of America; 6 Centre De La Science De La Biodiversité Du Québec, Université Laval, rue de l’Université, Québec, QC, Canada; 7 Canadian Wildlife Service, Environment and Climate Change Canada, Quebec, Quebec, Canada; National Museums of Kenya, KENYA

## Abstract

The ongoing global outbreak of Highly Pathogenic Avian Influenza Virus (HPAIv) Clade 2.3.4.4 H5N1 in poultry, which was first detected in the United States (USA) in February 2022, underscores the importance of improving food biosecurity and wild bird surveillance. The Delmarva Peninsula is vital to wintering waterfowl and to poultry production, thereby increasing the risk of HPAIv outbreaks. By using fine-scale GPS tracking of Greater Snow Geese (GSGO, N = 59) and Canada Geese (CANG, N = 9) over four winters (2019–2023), we 1) demonstrate a risk ranking system for poultry facilities based on exposure to wintering waterfowl, and 2) model this exposure in relation to temporal, geographic, and land cover factors. We showed that an ordinal-percentile ranking system, based on goose points per hour (gp/h) at poultry facilities, effectively predicted HPAIv H5N1 outbreak risks in the Delmarva Peninsula. Seven facilities with outbreaks were in the 78–99th risk percentile, including one ranked among the top 25 of 6,021 facilities. Such ranking criteria could be used to guide biosecurity efforts when response teams’ resources are limited. Notably, one-third of Delmarva poultry facilities had GPS-marked waterfowl present during winters, indicating a significant presence despite our limited sample sizes. We analyzed the variability in goose exposure rates near poultry facilities over four winters. Time (week) during winter was the key predictor of exposure, with greater GSGO presence in mid-late winter (Dec 15 to Feb 28) and CANG exposure peaking in early winter (Nov 28 to Dec 11), then stabilizing until late winter (Feb to Mar 13), whereupon exposure increased again. Colder temperatures (below 0°C) significantly increased GSGO exposure as they sought waste grain for energy. Goose exposure for both species was high throughout the winter, necessitating 24-hour surveillance. Proximity to sewage treatment plants (≤2 km), National Wildlife Refuges (≤5 km), water bodies (≤5 km), and the coast (≤20 km) increased exposure levels. Additionally, inland areas with features such as reservoirs and lakes supported large numbers of geese near facilities.

## Introduction

Avian influenza viruses (AIVs) are Type A influenzas of the Orthomyxoviridae family that have historically evolved with wild waterbird taxa such as *Anseriformes* (ducks, geese) and *Charadriiformes* (shorebirds, seabirds), causing limited to no clinical signs, hence the term low-pathogenic avian influenza virus (LPAIv). In recent history, these viruses have evolved as they are transmitted from wild birds to dense poultry populations [1,2], where they can mutate into a highly pathogenic form (HPAIv) causing high mortalities in chickens and turkeys [3]. HPAIv outbreaks can lead to significant poultry losses due to both high virulence and culling measures [4–6]. The 2014–2015 outbreak in the USA resulted in about 50 million domestic birds being lost [7], while the ongoing 2021–2026 outbreak has resulted in more than 206 million losses to date [8].

AIVs can be transmitted across individuals or populations of competent host reservoirs (e.g., dabbling ducks, waterbirds) via the fecal to oral route as infected birds feed and defecate in shared aquatic environments [1,4,9,10] or congregate close enough for bird-to-bird transmission [11]. Aquatic conditions related to temperature, salinity, and pH can affect how long AIVs can persist outside of their host, ranging from minutes to months [12–14]. Persistence relies on the presence of competent hosts at some point during the year [1,10,14–16].

Waterfowl play an important role in the preservation and spread of HPAIv over vast distances [17]. The Atlantic Flyway, stretching 4,800 km from the Arctic to the Caribbean, serves as a critical route for waterfowl and shorebirds, facilitating potential transcontinental HPAIv exchange [12,18,19]. Some species of waterfowl may shed HPAIv asymptomatically for up to 1–8 days, traveling distances of up to 3,000 km [20]. Waterfowl enable viral transfer through proximity, shared habitats, and high defecation rates [4,9,21–23].

HPAIv outbreaks in poultry facilities are linked to the presence of wild waterfowl [6,24,25]. Waterfowl near poultry farms increase the likelihood of HPAIv introduction, especially large flocks (≥500,000 birds) [26–28]. Cultivating feed crops attracts geese, raising HPAIv risk [29,30]. Run-off ponds nearby also enhance habitat value for waterfowl, increasing the introduction risk [31,32]. Waterbody contamination by waterfowl excrement adds to this risk [23,33]. Ducks and geese are carriers of HPAIv [2], with ducks favoring flooded areas, while geese, which are more versatile in habitat choice, could pose a greater exposure risk to poultry due to their feeding habits [34].

Advances in GPS animal tracking and biologging have greatly increased understanding of waterfowl movement, habitat use, migration, and behavioral ecology [35–38]. Despite these advances, relatively few studies have leveraged these technologies to address questions related to waterfowl disease ecology, particularly in the USA [7,17,24,28,39,40]. In particular, understanding the circadian activity of waterfowl near poultry farms in densely populated areas such as the Delmarva Peninsula (hereafter, DP) remains an information gap.

Using high-frequency GPS data on wintering Greater Snow Geese (*Anser caerulescens atlanticus*, hereafter GSGO) and Canada Geese (*Branta canadensis,* hereafter CANG) from 2019 to 2023, we assessed the exposure risk of poultry facilities to HPAIv. Our primary goal was to identify factors that lead to higher goose densities near poultry farms. We hypothesized that: goose activity and potential exposure risk to poultry facilities would be influenced by several environmental and spatial factors: 1) wintering geese would increasingly move inland toward poultry operations as coastal food sources become depleted; 2) facilities located adjacent to farm ponds would be particularly attractive to geese; 3) goose densities would remain consistent near farms during both day and night; 4) facilities closer to the coast and National Wildlife Refuges would experience higher exposure risk; 5) the presence of nearby anthropogenic or natural roosting sites would increase the likelihood of goose presence; 6) farms situated in landscapes with greater coverage of crops and wetlands would experience higher levels of goose activity; and 7) infected poultry facilities would correspond to higher modeled exposure risk rankings. These hypotheses aim to enhance our understanding of the interactions between wild waterfowl and commercial poultry in this economically significant region.

## Materials and methods

### Study area

**Fig 1:**
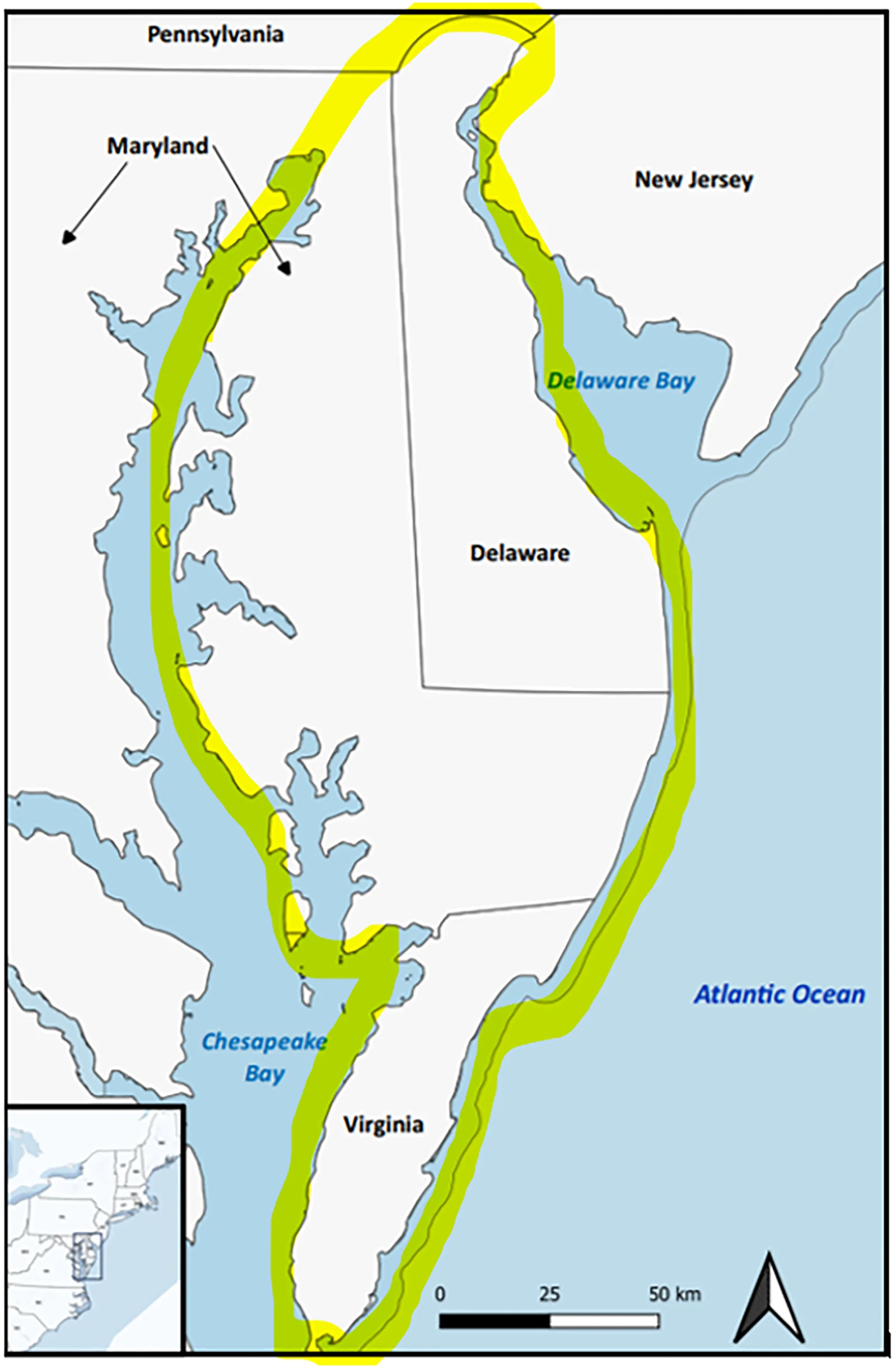
Delmarva Peninsula study area. Delmarva Peninsula study area (highlighted in yellow) within the larger Mid-Atlantic region (inset). Basemap imagery from OpenStreetMap (OSM).

The DP (Fig 1) served as the study area. Located along the Atlantic coast of the eastern USA, the DP comprises all of Delaware, the Eastern Shore of Maryland, and the southern tip of Virginia. It spans approximately 274 km long and 114 km wide, covering 15,540 km² (1.55 million ha) with a coastline of 5,180 km. Bordered by Chesapeake Bay to the west and the Atlantic Ocean to the east. The flat topography and sand-dominated soil keep elevations below 31 m throughout the region. The DP is an essential part of the Atlantic Flyway, featuring around 526,000 ha of important bird areas, including 243,000 ha of global significance [41].

The region’s climate is influenced by the Atlantic Ocean and the Chesapeake Bay, with an average annual temperature of 13.5°C and an average annual precipitation (mostly rainfall; infrequently snow) average of 112 cm [42,43]. Average low temperatures (2°C) occur in January–February, and average highs (25°C) in July. Agricultural land occupies about 43% of the area, including ~647,500 ha of farmland with ~6,000 commercial poultry farms (mostly for meat production) [44,45]. Dominant crops are corn, soybeans, and small grains. Other land types include wetlands (~688,000 ha, with 50% lost to agriculture [46]) and forests (~182,000 ha) [45].

### Trapping & telemetry

CANG were captured at migratory staging areas from November to March 2020–2022 and in June 2021. GSGO were captured at staging areas during May–June 2020–2022. Both species were fitted with Ornitela Ornitrack-N44 GPS/GSM neck collars (~45 g; Ornitela, UAB) using various capture methods, including swim-in and corral traps [47] and via rocket netting [48]. GSGO were captured at Île aux Oies, Quebec (47°08 N, 70°29 W) in collaboration with the University of Laval, while CANG were captured at various sites in Delaware and Maryland, primarily using baited traps. CANG captures involved checking traps twice daily and employing corral traps during their molt in June. All capturing and GPS attachment procedures were approved by the University of Delaware Animal Use and Care Committee (#1369), Federal Bird Banding Permit (#06961), the Comité de Protection des Animaux de l’Université Laval (CPAUL # 2019−304, VRR-19–010), and the Canadian Bird Banding Office (permit #10648). Further details on capture locations and techniques are in the S1 Table.

### GPS settings, configuration, and data processing

Solar-powered GPS tracking devices recorded locations every 5 minutes with <5 m error under sufficient solar charge. Data collected included UTC timestamps, coordinates (latitude/longitude; WGS 84), altitude, speed, directional degrees, and accelerometry values. Data were transmitted from the tags to the manufacturer for decoding and initial processing, then transmitted to Movebank repositories [49] for storage and secondary processing. Movement data retrieved from Movebank were analyzed using the R ‘move’ package [50,51]. Coordinate reference systems for GPS points and poultry facilities [44] were projected to UTM zone 18N for analysis.

#### Poultry facility locations within the Delmarva Peninsula.

We obtained commercial poultry facility locations within the DP from 2016–2017 [44]. We identified individual poultry facilities (N = 6,021) in the dataset by aerial imagery (4,900 confirmed by the Delmarva Chicken Association [52], and 1,121 private/retired/other). Facility locations were documented; however, facility ownership, HPAIv infection status/history, and other personal information were redacted. We converted each poultry facility polygon to a line shapefile type to calculate accurate distance from the facility perimeter (rectangular polygon), rather than the centroid.

#### Waterfowl – Poultry facility proximity and exposure analysis.

Our GPS dataset of 7,621,765 locations was filtered for winter (1 November–15 March 2020–2022) and limited to points within the terrestrial bounds of the DP. Flight points were excluded, focusing on ground points for better habitat selection analysis. We converted the GPS points into tracks using the ‘amt’ [53] and ‘moveHMM’ [54] packages in R, classifying points as flight if their step speed exceeded 5 km/h [55].

To calculate proximity to poultry facilities, we measured distances from goose GPS ground points to the nearest DP poultry facility perimeter. We counted the number of GPS locations occurring within 2 km of each facility perimeter. This threshold was selected because it approximated the average flight distance observed among marked geese in this study and represented a biologically relevant zone within which an average goose could potentially reach a facility during a single movement event. Therefore, locations within 2 km were interpreted as indicators of potential exposure opportunity rather than direct facility use. We calculated cumulative goose exposure time in “goose exposure days” by multiplying the number of points by the 5-minute interval between GPS collection and converting to days. Goose exposure days were calculated as the cumulative time spent by all tracked geese near poultry facilities and therefore represent total goose-use time rather than exposure averaged across individuals. For modeling exposure at facilities, we used this ‘goose points per hour’ (gp/h) metric to calculate hourly exposure rates at each facility. We acknowledge that a GPS location 10 m from a facility likely represents a different level of potential interaction than one located 1,900 m away. Our objective, however, was not to estimate direct contact risk as a continuous function of distance, but rather to quantify the relative frequency with which geese occurred within a biologically relevant zone surrounding poultry facilities. We selected a 2-km threshold because it approximated the average flight distance observed among marked geese in this study and represented a distance over which an average goose could theoretically move and reach a facility in a single flight. Thus, the metric was intended to represent potential exposure opportunity rather than direct facility use.

#### Assessing GPS data at Delmarva facilities with HPAIv detections since outbreak.

There were 7 HPAIv outbreaks at DP commercial poultry facilities (DE = 3, MD = 4) between 22 February –18 March 2022, resulting in mortality (primarily via culling) of ~3.2 million poultry [8]. We compared mean goose exposure between poultry facilities with HPAIv outbreaks and those without reported outbreaks. We buffered the first day of reported detection within the DP (22 February 2022) by 10 days to reflect the duration of waterfowl exposure that precedes HPAIv outbreak [56]. We therefore made our sampling period a 34-day window from 12 February–18 March 2022. To determine if HPAIv-infected facilities experienced greater than average goose exposure, we examined the percentile rankings of goose exposure days for infected farms relative to the distribution of goose exposure days of all farms within the DP for both the 34-day outbreak window and since the first North American detection in a wild bird (12 January 2021).

#### Modeling waterfowl – Poultry proximity.

Distance to coast was determined using TIGER/Line data U.S. Census Bureau, and water bodies were determined using USGS datasets for farm ponds [57] and larger waterbodies, as well as the coast (USGS National Hydrography Dataset) [58]. Sewage treatment facility locations came from HydroSHEDS [59]. We also derived distances to National Wildlife Refuges (hereafter NWRs) from the USGS Protected Area Database [60].

Additional covariates included the summation of the number of adjacent farm centroids within a 2-km buffer to assess any potential effects of farm clustering. Hourly mean temperatures were computed using European Centre for Medium-Range Weather Forecasts (ECMWF) Reanalysis version 5 (ERA5) data [61]. We used the 2019 National Land Cover Data dataset [62] to compute proportion of 10 land cover types (open water, developed [all types consolidated], barren, grassland, forest [all types consolidated], shrub/scrub, hay/pasture, crops, emergent wetland, and woody wetland) at the landscape scale (2-km radius around farms). The time of day was calculated using the ‘suncalc’ package in R [63] to reference `day` (sun above the horizon) vs `night` (sun below the horizon) condition for each bird location at the time of collection.

**Fig 2:**
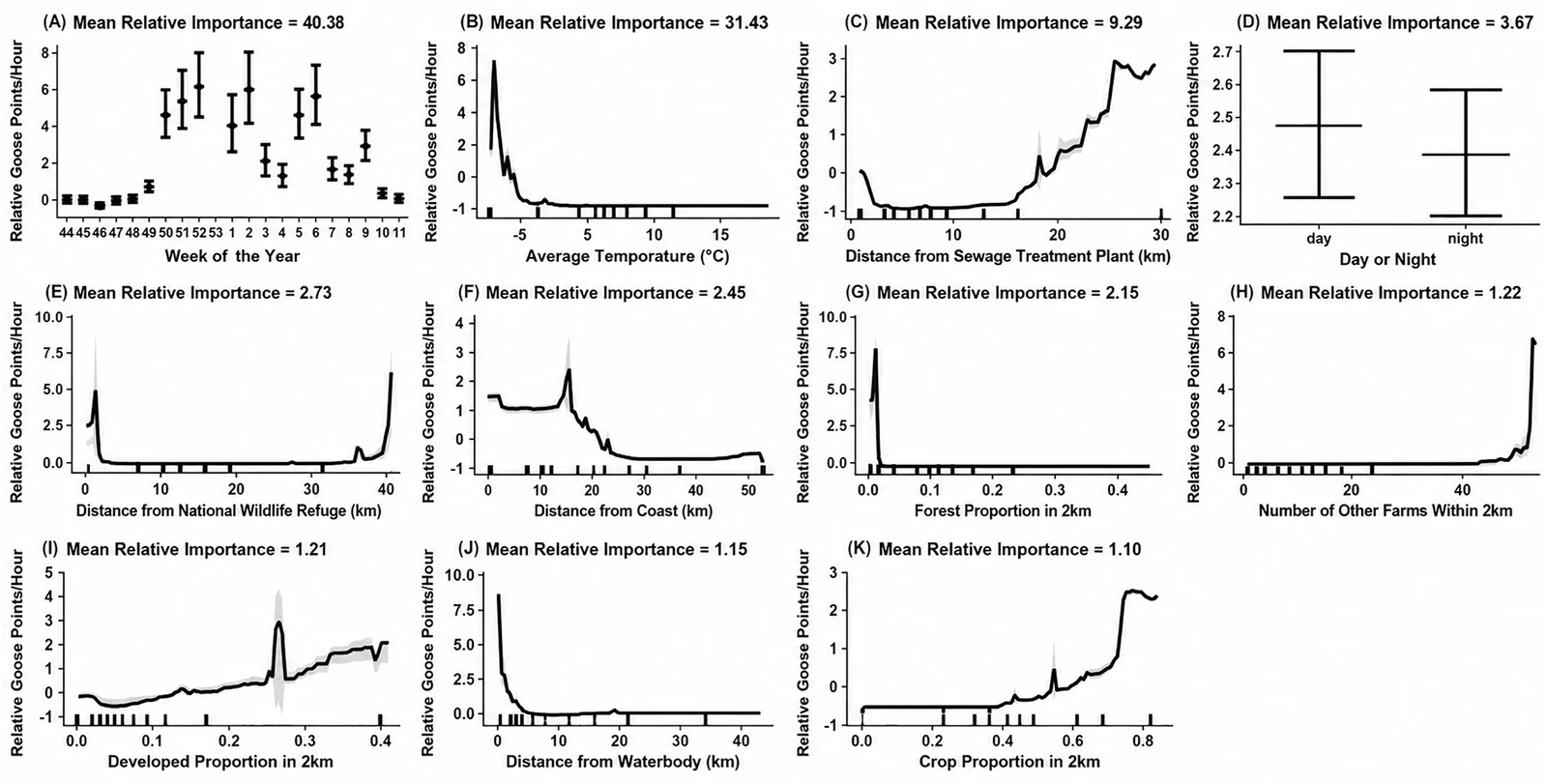
Relative importance of environmental predictors of Greater Snow Goose use at Delmarva poultry facilities. Mean relative Greater Snow Goose points per hour at Delmarva Peninsula poultry facilities as a function of week of the year (A), average temperature (B), distance to geographic features (C, E-F, and J), time of day (D), number of adjacent farms (H), and land cover (G, I, and K) across 25 boosted regression tree models across 4 winters (1 November – 15 March 2019–2023). Shading represents SE of mean response among 25.

**Fig 3:**
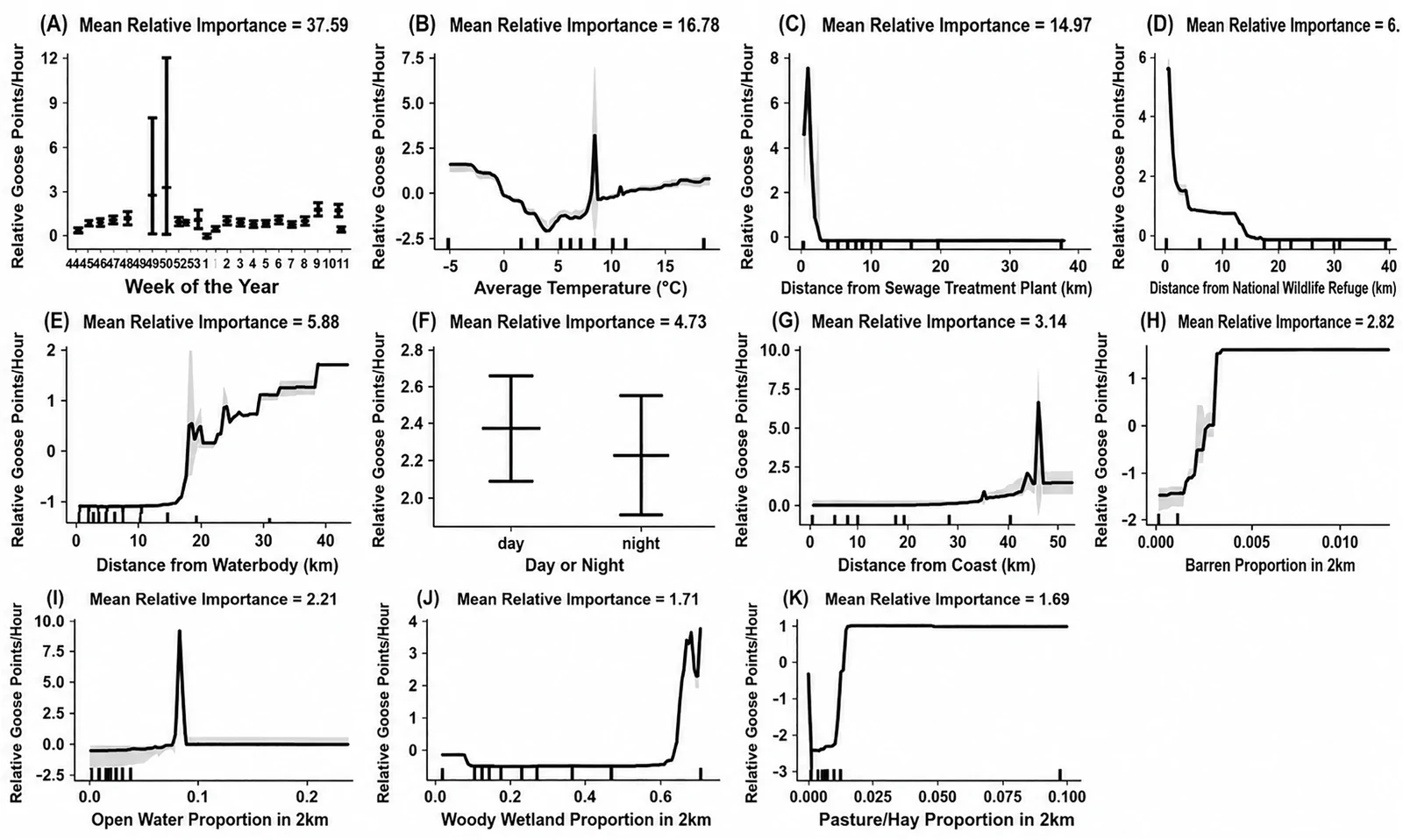
Relative importance of environmental predictors of Canada Goose use at Delmarva poultry facilities. Mean relative Canada Goose points per hour at Delmarva Peninsula poultry facilities as a function of week of the year (A), average temperature (B), distance to geographic features (C-E and G), time of day (F), and land cover (H-K) across 25 boosted regression tree models across 4 winters (1 November – 15 March 2019–2023). Shading represents SE of mean response among 25 models. Error bars (whiskers) indicate ±1 SE.

**Fig 4:**
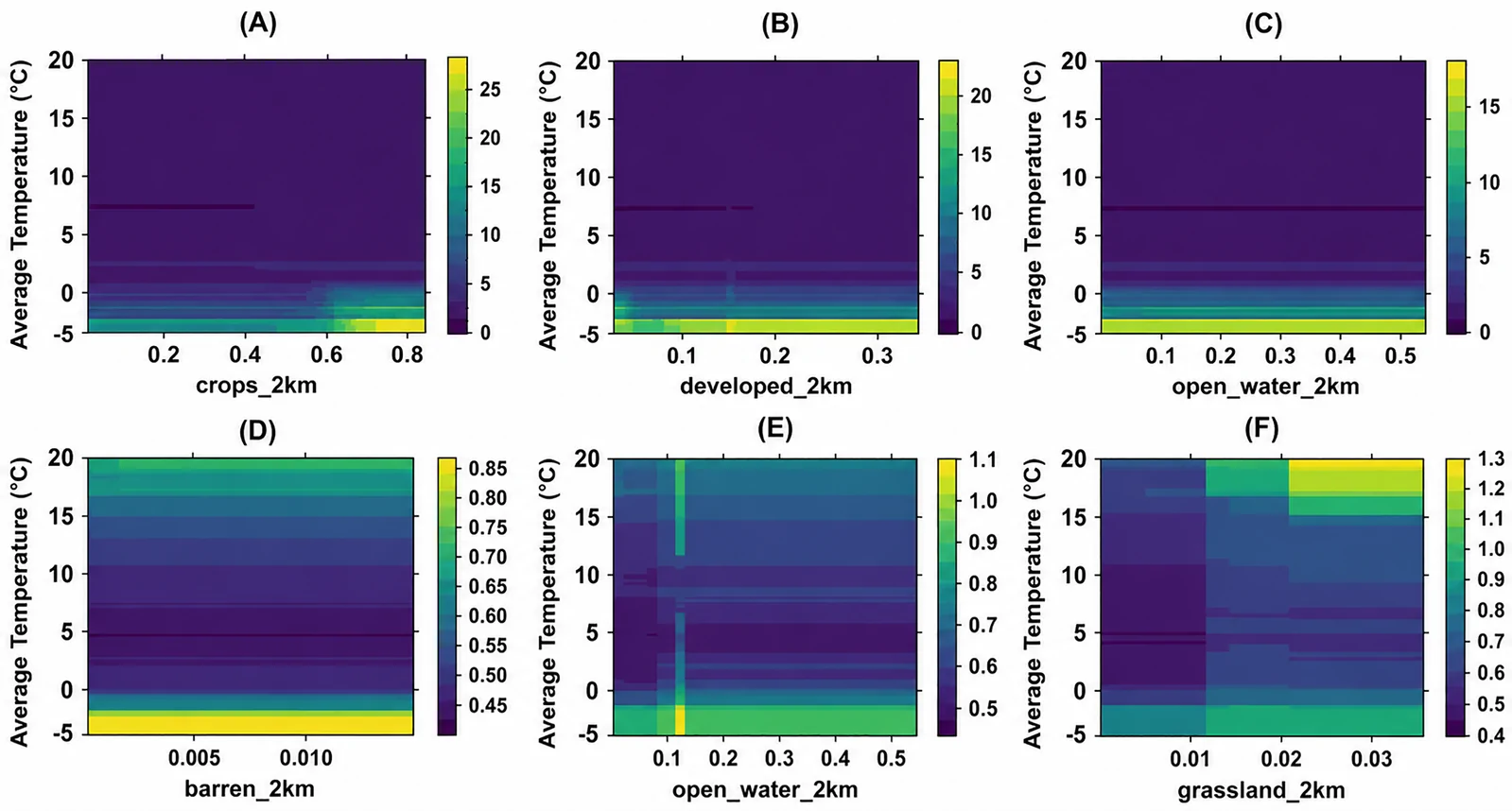
Interactive effects of temperature and land cover on goose exposure at poultry facilities. Interactive effects of average temperature and landcover types on poultry facility exposure (goose points per hour) to Greater Snow Goose (A-C) and Canada Goose (D-F) across 25 Boosted regression tree models across 4 winters (1 November – 15 March 2019–2023).

**Fig 5:**
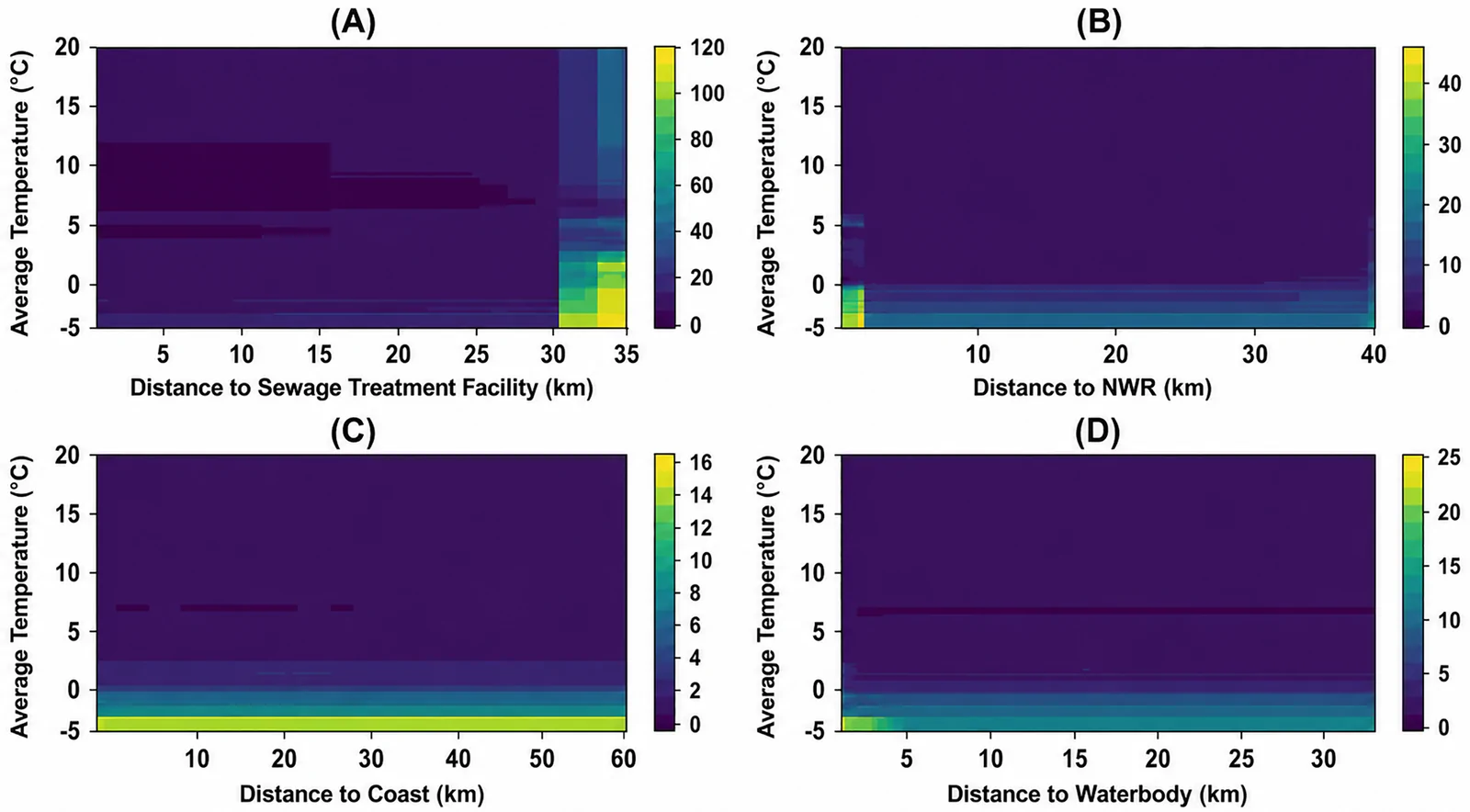
Interactive effects of temperature and geographic features on Greater Snow Goose exposure at poultry facilities. Interactive effects of average winter temperature and geographical features on poultry facility exposure (goose points per hour) to Greater Snow Goose (A-D) across 25 boosted regression tree models across 4 winters (1 November – 15 March 2019–2023).

**Fig 6:**
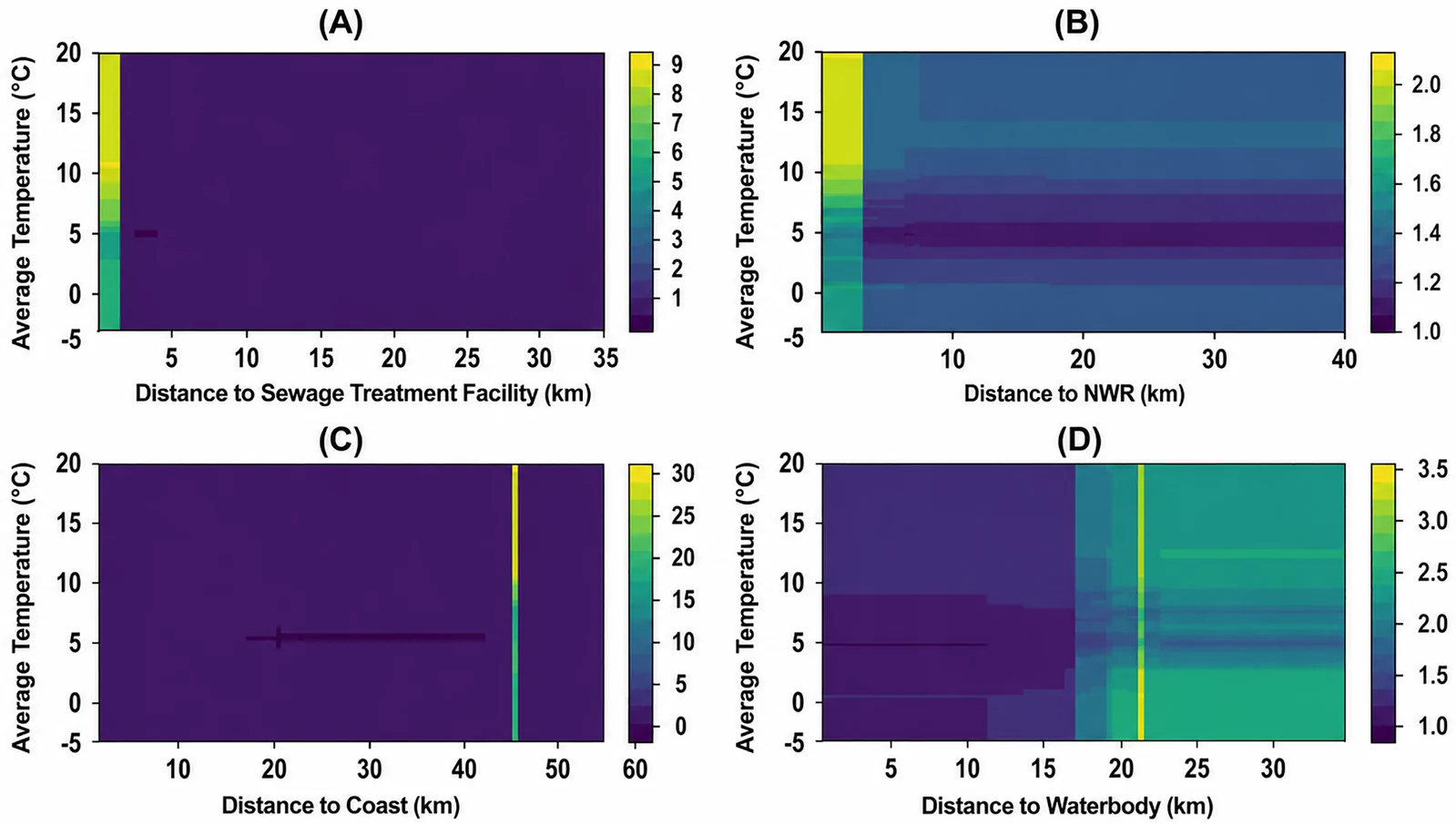
Interactive effects of temperature and geographic features on Canada Goose exposure at poultry facilities. Interactive effects of average winter temperature and geographical features on poultry facility exposure (goose points per hour) for Canada Goose (A-D) across 25 boosted regression tree models across 4 winters (1 November – 15 March 2019–2023).

We modeled farm exposure for GSGO and CANG around poultry facilities (50 GSGO [200,951 locations] and 7 CANG [162,919 locations]) using Boosted Regression Trees with 19 predictor variables. Variables included distances from key features (Atlantic coast, sewage treatment plants, NWR, and water bodies), landscape land cover proportions, daily mean temperatures, time of day, and facility characteristics (e.g., number of adjacent farms). To reduce spatial autocorrelation, we partitioned grid cells into 25 groups such that grid cells within a given group were separated by at least 5 km. Modeling iterations were then conducted separately for each group, and covariate data were combined across years. We used a complete pairwise test to evaluate collinearity among predictors. We modeled gp/h separately for CANG and GSGO and ran 25 modeling iterations (one for each grid partition) for each species using the ‘gbm.step’ function in the ‘*dismo’* package [64] in R and averaged predictions to obtain mean goose detections per hour for each facility. The relative importance of predictors (Tables 2 and 3) was assessed based on their effectiveness in splitting data across the ensemble of Boosted Regression Trees to assign an importance rank [65]. To facilitate assessment of modeled relationships between each predictor variable and exposure (gp/h), we averaged effects of all other modeled predictors and centered relationships on the model’s average prediction (Figs 2–6, S2 Fig). We could have used an alternative framework involving mixed-effects models that account for repeated observations from the same individuals through random effects. In such models, inference is based on movement behavior while accounting for within-individual correlation. Instead, we fit models using unaggregated, individual-level observations to maintain data granularity and preclude any ecological fallacy resulting from cross-level inference [66]. Although hierarchical or temporal data aggregation typically yields higher goodness-of-fit metrics by stripping out within-group variance [67], it masks critical individual stochasticity and unobserved behavioral heterogeneity [68,69]. By retaining the full scope of variation within and across individuals, we can more precisely assess why an individual goose is near a farm, without the potential distortion from population-level aggregation. Thus, the lower deviance explained reported herein is a structural mathematical consequence of our modeling choice to retain disaggregated variance components, rather than a failure of the model to capture meaningful underlying patterns. This approach ensures unbiased parameter estimates and maintains the ecological validity of the inference.

## Results and discussion

### Distributions of wintering geese in the Delmarva Peninsula and poultry facilities

**Fig 7:**
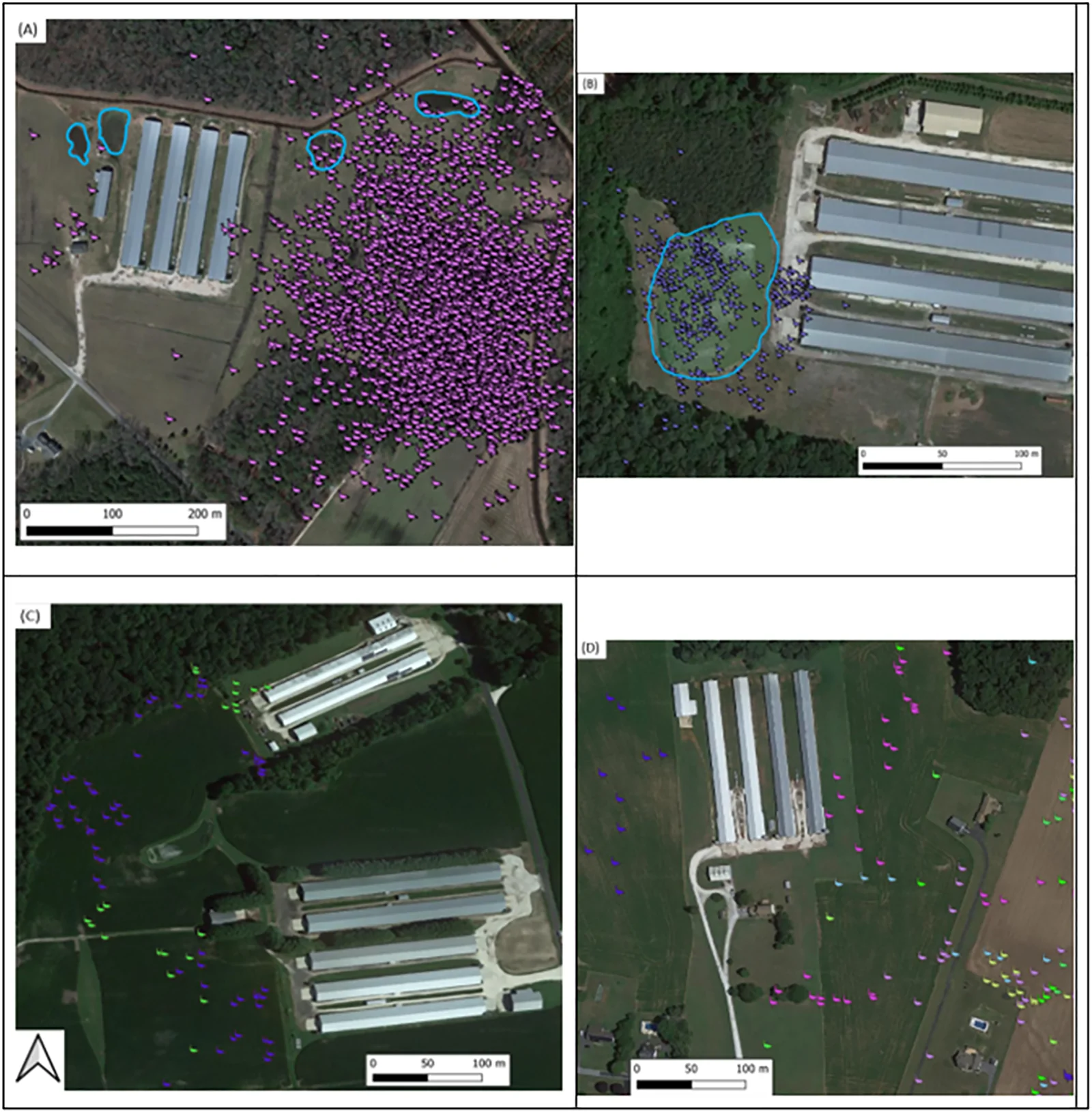
Examples of poultry facility exposure by GPS-marked Canada Geese (CANG) and Greater Snow Geese (GSGO). Four cases of poultry facility exposure and adjacent property use by wintering Canada Geese (CANG) and Greater Snow Geese (GSGO) in the Delmarva Peninsula during the winter of 2022-2023 (A-D). Two cases included facilities associated with farm ponds (A-B) (blue outline). Each colored point represents 5 minutes of exposure time for a distinct individual (A = CANG, n = 1, purple; B = GSGO, n = 1, blue; C = GSGO, n = 2, yellow/blue; D = GSGO, n = 4, blue/pink/green/yellow). Background imagery from the U.S. Department of Agriculture National Agriculture Imagery Program (NAIP).

GSGO (590,295 locations among 59 marked individuals) were found in the greatest concentrations at coastal sites in Delaware; however, high concentrations of birds were also found inland within agricultural sites on the border of Maryland and Delaware (S1 Fig-B). Overlap between wintering geese and poultry facilities was most apparent at inland sites throughout the winter (S1 Fig). CANG (542,978 locations among 9 individuals) were found both within coastal and inland sites within the DP (S1 Fig-C). In addition to refuges and rivers, inland sites for CANG consisted of urban ponds, creeks, medium-large lakes, sewage treatment facilities, and sand/gravel pits, mostly adjacent to agriculture. We found that the average distances to a poultry house for GSGO and CANG were 3.61 km ± 0.003 km and 4.57 km ± 0.004 km, respectively, and the minimum distances were 1.7 m and 3.9 m, respectively (Fig 7).

One third (2,012) of all DP poultry facilities had GSGO within a 2 km buffer around each farm during all winters (2019–2023). Throughout all winters combined, 50 out of 59 (84.8%) GSGO were present within a DP facility buffer. Across all winters, January had the greatest number of GSGO detections (48.3%). GSGO were 3.33% ± 0.01% more likely to be within DP farm buffers during the day than the night (ꭓ = 445.71, df = 1, p < 0.001). The semi-monthly (i.e., half-month) period which had the most GSGO detections within farm buffers was 16–31 January. We found that 38.6% (2,330) of farms in the DP had at least one GPS-marked GSGO or CANG on the ground within a buffer of the facility. We found that 7.1% (429) of all DP poultry facilities had CANG detected within a buffer during all winters (2021–2023). Across all winters combined, 7 out of 9 (77.8%) GPS-marked CANG were present within at least one DP farm buffer. November had the greatest number of CANG detections (25.6%); however, this was not significantly different from December or February. CANG were 0.43% ± 0.01% more likely to be near farms during the day than the night (ꭓ = 5.913, df = 1, p = 0.015, α = 0.01). We found that for all winters, the bimonthly period that had the most CANG detections near farms was 1–15 March. There was a total of 13,577 goose hours of CANG exposure (647 hours/individual/winter) at DP poultry facilities across all winters (2021–2023) and a total of 16,745 goose hours of GSGO exposure (67 goose exposure hours/individual/winter) for GSGO. We found that 1.8% (108) of all DP poultry facilities had both GPS-marked CANG and GSGO within their buffer during each winter.

**Fig 8:**
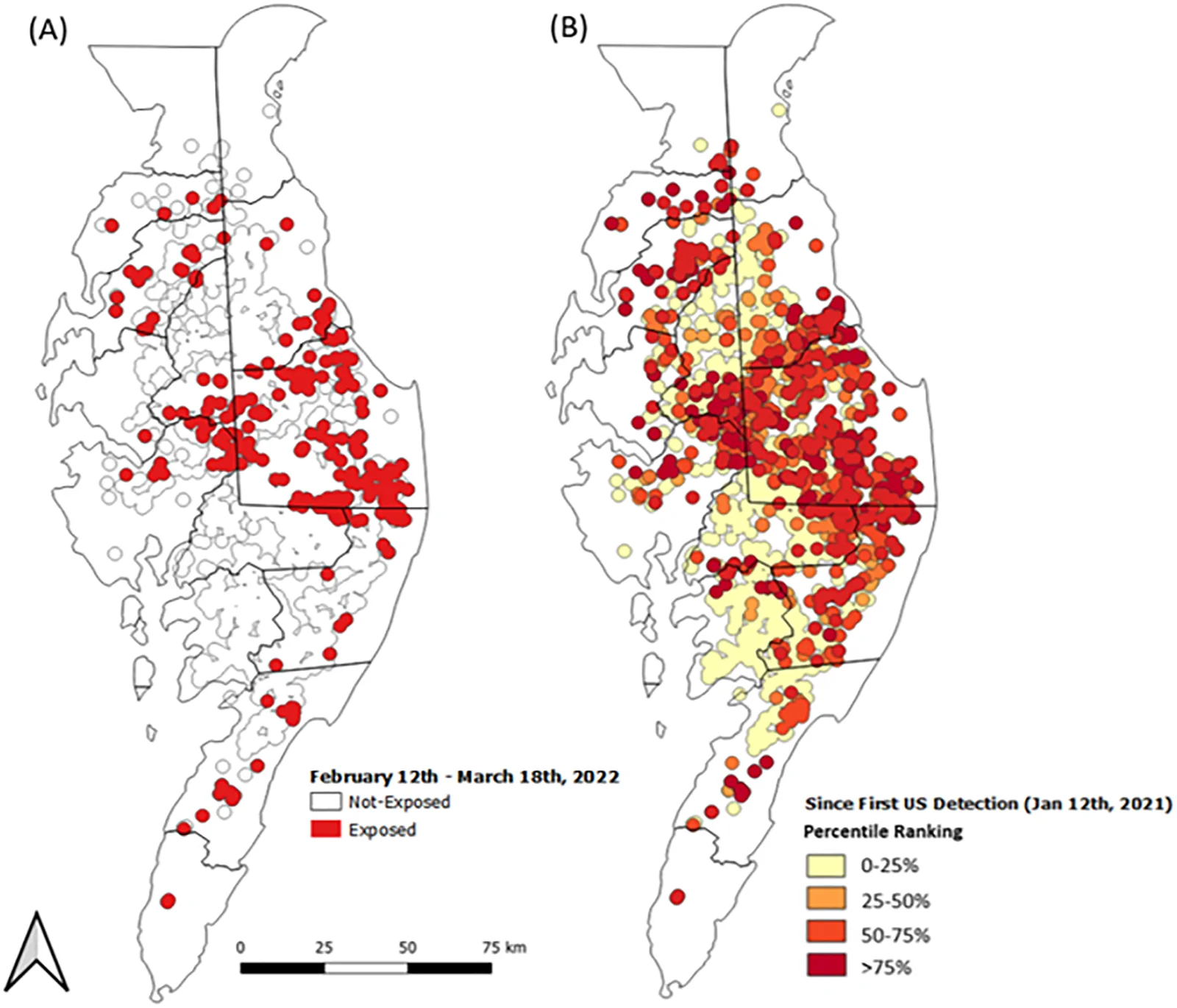
Poultry facility goose exposure and historical exposure rankings during the 2022 H5N1 outbreak on the Delmarva Peninsula. Map of A) poultry facility exposure within 2-km of GPS-marked Canada Geese and Greater Snow Geese during the 34-day period (12 February 2022 − 18 March 2022) of HPAIv cases at Delmarva Peninsula facilities, and B) percentile rankings of goose exposure days within 2 km of poultry facilities since the first USA wild bird detection of HPAIv on 12 January 2021. U.S. Census Bureau TIGER/Line administrative boundaries.

During the 34-day sampling window of the 2022 H5N1 outbreak (12 February 2022 - 18 March 2022), 11.5% (692) of farms in the DP were exposed to GPS-marked waterfowl (n = 20) within a buffer of the facility. GSGO were within the buffers of 10.1% (606) of all DP poultry facility buffers during the DP outbreak window. Fifteen out of 59 (25.4%) GPS-marked GSGO were present within a DP farm buffer during the DP outbreak window, which represented 1,203 combined goose hours, or 80 goose exposure hours per individual for the 34-day window. CANG were within the buffers of 1.4% (86) of all DP poultry facility buffers during the outbreak. Five out of nine (55.6%) GPS-marked CANG were present within a DP farm buffer during the DP outbreak window, representing a combined 1,605 goose hours, or 321 goose exposure hours per individual, over the 34-day window. None of the DP poultry farms had both GPS-marked geese present within the same buffer during the 34-day window. The average cumulative exposure across all DP poultry facilities with at least one instance of exposure within a buffer (n = 692) during the HPAIv outbreak window was 1.52 goose days (36.5 goose hours). Maximum cumulative exposure at any facility was 19.8 goose days (475 goose hours). In the 34-day window, GPS-marked waterfowl were detected at one of seven confirmed HPAIv-infected DP poultry facilities (Table 1). At this facility, there were 19.7 goose hours (2.4% of the infection period window) of exposure by two GSGO during the period spanning 14–18 February 2022. This infected facility was also within the 99^th^ percentile of risk for total goose exposure days since the first H5N1 wild bird detection in the USA (Table 1). We detected no GPS-marked CANG at any of the seven infected sites during the DP outbreak window, or since the first wild bird detection (Table 1). All seven H5N1-infected facilities were exposed to GSGO since the date of the first HPAIv wild bird detection in the USA. All seven facilities infected with HPAIv in the DP were ranked within the top 25% priority farms (Fig 8.), with one infected facility being one of the top-25 facilities flagged based on risk (i.e., top 0.4%). The central region of the DP had the greatest relative exposure of poultry farms to GPS-marked waterfowl (Fig 8 and S1 Fig.).

**Table 1:** Goose exposure at Delmarva Peninsula poultry facilities during the 2022 H5N1 outbreak period.

Farm ID	Exposure during 34-Day window (12 February 18 March 2022)			Exposure since 12 January 2021		
	GSGO hours (geese)	CANG hours (geese)	Exposure	GSGO hours	CANG hours	Percentile rank
1	0	0	Not-Exposed	54.5	0	89^th^
2	0	0	Not-Exposed	36.7	0	85^th^
3	0	0	Not-Exposed	18.2	0	80^th^
4	0	0	Not-Exposed	34.8	0	85^th^
5	0	0	Not-Exposed	86.4	0	91^st^
6	0	0	Not-Exposed	13.9	0	78^th^
7	19.7 (2)	0	Exposed	1421.3	0	99^th^
TOTAL	19.7	0		1665.8	0	

Poultry facility exposure to GPS-marked Greater Snow Geese (GSGO) and Canada Geese (CANG) within 2 km buffers during the 34-day period (12 February – 18 March 2022) of H5N1 cases at Delmarva Peninsula facilities, and percentile ranking based on GPS-marked geese detections within 2 km of a poultry facility since the first USA wild bird detection of H5N1 on 12 January 2021.

### Modeling waterfowl – Poultry exposure

We modeled variability in the cumulative bird hours for the 50 GSGO (200,951 locations) and 7 CANG (162,919 locations) within facility buffers over four winters (2019–2023). The spatial dependence of goose exposure time at poultry farms was determined by fitting an exponential curve to the semivariance of goose exposure time, with a maximum lag distance of 12 km and a range parameter of 4,384 m. We subsampled poultry farm locations by 4.5 km based on this.

The cross-validated deviance explained by models of observed gp/h at DP poultry farms was 16.68 ± 0.5% (mean ± SE) for GSGO and 25.17 ± 2.65% (mean ± SE) for CANG. The top three biologically relevant predictors for both species were week of the year, average daily temperature, and distance from sewage treatment facility (Tables 2 and 3). The effect size of top predictors varied by species; however, in general, the temporal period (week) had the greatest mean relative importance, followed by average temperature. In general, geographical covariates (distance to features) and landscape-scale land cover composition ranked comparatively lower at predicting gp/h at poultry farms. All predictor response plots with mean relative response values <1.0 are not reported in the results but can be found within S2 Fig, ordered by mean response for each species.

**Table 2:** Relative importance of boosted regression tree model predictors of Greater Snow Goose activity at Delmarva Peninsula poultry facilities during four winters (2019–2023).

Predictor	Mean importance	Rank
Week of the year	40.38	1
Average daily temperature (°C)	31.43	2
Distance from sewage treatment facility (km)	9.29	3
Time of day (day or night)	3.67	4
Distance from National Wildlife Refuge (km)	2.73	5
Distance from coast (km)	2.45	6
Forest proportion at 2 km	2.15	7
Adjacent farms within 2 km	1.22	8
Developed proportion at 2 km	1.21	9
Distance from waterbody (km)	1.15	10
Crop proportion at 2 km	1.10	11
Open water proportion at 2 km	0.76	12
Barren proportion at 2 km	0.61	13
Shrub/scrub proportion at 2 km	0.45	14
Woody wetland proportion at 2 km	0.44	15
Pasture/hay proportion at 2 km	0.41	16
Emergent wetland proportion at 2 km	0.30	17
Farm pond within 500 m	0.12	18
Grassland proportion at 2 km	0.12	19

Relative importance of boosted regression tree modeled predictors of Greater Snow Goose points/hour (gp/h) at Delmarva poultry farms for four winters (1 November – 15 March 2019–2023). Mean cross-validated deviance explained by the models was 16.68 ± 0.5% (mean ± SE).

**Table 3:** Relative importance of boosted regression tree model predictors of Canada Goose activity at Delmarva Peninsula poultry facilities during four winters (2019–2023).

Predictor	Mean importance	Rank
Week of the year	37.59	1
Average daily temperature (°C)	16.78	2
Distance from sewage treatment facility (km)	14.97	3
Distance from National Wildlife Refuge (km)	6.14	4
Distance from waterbody (km)	5.88	5
Time of day (day or night)	4.73	6
Distance from coast (km)	3.14	7
Barren proportion at 2 km	2.82	8
Open water proportion at 2 km	2.21	9
Woody wetland proportion at 2 km	1.71	10
Pasture/hay proportion at 2 km	1.69	11
Grassland proportion at 2 km	0.78	12
Forest proportion at 2 km	0.48	13
Emergent wetland proportion at 2 km	0.26	14
Farm pond within 500 m	0.22	15
Developed proportion at 2 km	0.19	16
Crop proportion at 2 km	0.19	17
Shrub/scrub proportion at 2 km	0.12	18
Adjacent farms within 2 km	0.10	19

Relative importance of boosted regression tree modeled predictors of Canada Goose points/hour (gp/h) at Delmarva poultry farms for four winters (1 November – 15 March 2019–2023). Mean cross-validated deviance explained by the models was 25.17 ± 2.65%. (mean ± SE).

### Environmental and temporal variables

For GSGO, the average exposure across all weeks was 3.13 ± 0.29 gp/h. GSGO exposure was greater on average during weeks 52 (26 December – 31 January), 2 (10 January – 16 January), and 6 (7 February – 13 February) (Fig 2A). GSGO exposure was lowest on average during weeks 44–49 (31 October – 11 December), and 10–11 (7 March – 20 March), with detections generally lower than average (Fig 2A). Goose exposure was markedly higher with average temperatures ≤0°C. GSGO exposure was greatest at −1.86°C, at eight times the average exposure rate across all temperatures. GSGO exposure generally plateaued when temperatures approached 2.5°C (Fig 2B). GSGO gp/h was marginally higher during the daytime (2.46 gp/h) than during the night (2.36 gp/h, Fig 2D).

For CANG, the average exposure across all weeks was 2.35 ± 0.14 gp/h. CANG exposure was greater on average during weeks 48–49 (28 November – 11 December), albeit with high variation (Fig 3A). Weeks 9–10 (28 February – 13 March) also had slightly higher average relative rates of CANG exposure. CANG exposure was lowest during weeks 44 (31 October – 6 November), 53–1 (31 December – 9 Jan), and 11 (14 March – 20 March, Fig 3A). CANG exposure increased in a bimodal pattern, both when average temperatures fell below ~2.5°C and when they rose above 8°C (Fig 3B). CANG exposure was slightly higher during the daytime (2.34 gp/h) than during the night (2.18 gp/h, Fig 3D).

### Geographic location

GSGO exposure slightly increased at distances ≤1 km from sewage treatment facilities but strongly increased between 15–25 km (Fig 2C). Colder temperatures (≤2.5°C) increased the effect of treatment facilities on GSGO exposure at this range (Fig 5A). GSGO exposure was generally greatest between 0–17.5 km from the coast and declined when distances from the coast were ≥20 km, with the greatest GSGO exposure being observed at distances ~16 km from the coast (Fig 2F). GSGO exposure was higher in temperatures ≤0°C regardless of distance to the coast (Fig 5C). There was a bimodal relationship between distance from a NWR and GSGO exposure, such that rates of exposure ~5–6 times greater were observed when distances from a NWR ranged between 0.3–1.5 km and 39–40 km (Fig 2E). Decreasing temperatures increased the effect of GSGO exposure at distances <2.5 km from a NWR (Fig 5B). There was a strong effect of decreasing distances from a waterbody (especially <2 km) on increasing GSGO exposure (Fig 2J). Maximum GSGO exposure was observed when a facility had a waterbody <5km, with facilities with a waterbody ≤0.5 km experiencing ~ 8 times the average response rate strength for this predictor variable. Decreasing temperatures increased the effect of GSGO exposure at distances <5 km from a waterbody (Fig 5D). Between types of waterbody, GSGO spent the greatest proportion of time near swamp/marsh (63 ± 5%), stream/river (35 ± 5%), and lake/pond (13 ± 2%); note percentages do not sum to 100% given mean estimates averaged across all birds’ proportions. The rate of facility exposure to GSGO increased as a function of an increasing number of adjacent farms within a buffer, particularly with >40 adjacent farms (Fig 2H).

There was a considerable effect of decreasing distance to a sewage treatment facility on CANG exposure. When the distance from a sewage treatment facility was < 2 km, average CANG exposure increased by 6–8 times (Fig 3C). CANG exposure fell considerably when distance from a sewage treatment facility was ≥ 2 km. The effect of sewage treatment facilities on CANG exposure was greatest in temperatures ≥9°C (Fig 6A). The greatest rates of CANG exposure were observed as a function of decreasing distance to a NWR, especially when distances were ≤3 km. Rates of CANG exposure were comparatively lower when distances from NWR > 10 km (Fig 3D). The effect of NWR on CANG exposure was greatest in warmer temperatures closer to NWR sites (Fig 6B). The highest rates of CANG exposure occurred with increasing distance from the coast, with maximum exposure observed at distance 40–45 km (Fig 3G), though with greater uncertainty. CANG exposure generally increased as a function of increasing distance from a waterbody, particularly with distances >15 km. Maximum CANG exposure was observed at distances approaching 40 km from a waterbody (Fig 3E). CANG spent the greatest proportion of time near streams/rivers (69 ± 16%), lakes/ponds (44 ± 15%), and swamps/marshes (14 ± 9%).

### Landscape composition

The greatest effect on GSGO exposure within buffers occurred when the proportion of forest was ≤ 5%, sharply declining as forest became more common (Fig 2G). GSGO exposure generally increased when >25% of land was developed (Fig 2I). GSGO exposure increased as a function of increasing crops. GSGO exposure increased exponentially when the proportion of crops exceeded 60%, and temperatures were ≤0°C (Figs 2K and 4A). GSGO exposure was greatest with developed landcover in temperatures ≤0°C (Fig 4B). The effect size of GSGO exposure with open water within buffers was always greater in temperatures <0°C (Fig 4C).

Relative CANG exposure increased substantially when barren land within buffers exceeded 0% and approached 0.5% (Fig 3H). The effect of barren land cover on CANG exposure was relatively weak and likely driven by temperatures <0°C (Fig 4D). CANG exposure increased as a function of increasing proportions of open water (Fig 3I). Maximum values of CANG exposure were observed when proportions of open water reached 14% (~9 times greater than values above or below). Proportions of open water >15% slightly increased CANG exposure relative to the rate of increase when open water was 0%. CANG exposure in relation to open-water proportions was about twice as high when temperatures were <0°C (Fig 4E). CANG exposure was greatest when proportions of woody wetland were between 60–75%, ~ 4 times the average response rate of lesser proportions (Fig 3J). There was a considerable increase in CANG exposure when the proportion of hay/pasture within buffers was 1.25–2.5%; however, they generally plateaued at proportions 2.5–10% (Fig 3K). The most notable interactive effect between temperature and land cover use by CANG was with grassland, such that CANG exposure increased with increasing proportions of grassland as a function of increasing winter temperatures (Fig 4F).

## Conclusions

As of June 23, 2026, there were 19,511 and 3,508 wild bird detections of confirmed HPAIv in the USA and Canada, respectively [70,71]. CANG and GSGO are considered species of moderate AI concern comprising 10.0% (1,949) and 6.0% (1,161) of all wild bird detections in the USA and 23.7% (831) and 9.4% (329) of detections in Canada [70,71], respectively. The first documented confirmed case of HPAIv (HPAIv clade 2.3.4.4 H5/H5N1 subtype) in CANG in the DP was submitted from a deceased wild bird found from this study. Our *a priori* selection of CANG and GSGO as target species was justified, given that the species ranked 2^nd^ and 3^rd^, respectively in total number of wild waterfowl detections [70]. Mallards ranked 1^st^ and were also chosen as a candidate species for the risk matrix, but limited samples excluded them from analysis.

Waterfowl near poultry facilities have been linked to HPAIv outbreaks [1,5,7,72,73]. Our study examined the spatial and temporal variability in waterfowl exposure rates close to high-production poultry farms and supports findings that waterfowl often visit these areas [7]. With GPS tracking of only a small fraction of populations (~1.2 million CANG and ~750,000 GSGO, [74]), many farms are likely visited by unmarked individuals, especially since these species travel in large flocks [75–77]. During waterfowl virus prevalence studies, positive AI cases were seasonally found in both GSGO (12%; seasonal range 0–22%) and CANG (4%; seasonal range 1–7%; [78–80].

Waterfowl exposure time near poultry facilities can be a key metric for predicting HPAIv outbreaks [17]. Goose exposure ranked much higher at HPAIv-infected facilities (each facility with the top 22^nd^ risk percentile) than at uninfected ones. This supports the idea that exposure frequency drives HPAIv transmission risk [5,6,28,81,82]. GSGO were found at one of the seven HPAIv-infected facilities during the 34-day outbreak period and at all seven facilities since January 12, 2021. CANG were not detected at any infected sites, but their interaction with GSGO (1.8% of farms had both species) could facilitate viral exchange between populations. Although our sample sizes were small relative to the total population size (CANG: N = 9, GSGO: N = 59), our marked individuals visited a notable percentage of facilities (7.1% of facilities visited by CANG, 33.3% for GSGO). Given the social behavior of both species, our findings may underestimate the actual exposure of farms to wintering geese because visual observations further confirmed that both species often travel in flocks.

In addition to exploring the cumulative exposure of waterfowl at winter facilities, we examined temporal variation in factors influencing goose proximity. We observed that some geese visit multiple sites for short periods, while others show longer-term site fidelity, which is significant as longer presence indicates greater HPAIv exposure risk [26,27]. For instance, CANG in metropolitan Chicago demonstrated strong site fidelity, potentially prioritizing sanctuary over food quality [83]. Longer exposure may impart greater transmission risk due to behavioral patterns that cause site fidelity, such as roost site selection to avoid disturbance, and the resulting concentration of biologic activity, such as defecation, into the surrounding environment, which may be the mechanism of transmission. CANG have high defecation rates, excreting up to 3.6 x 10^4 fecal coliforms per gram daily [23], and may deposit about 4% of their body weight in feces each day [2,84]. Seroprevalence studies indicate that ~14% of CANG eventually become exposed to HPAIv through water contamination, affected by season and the waterbody’s hydrodynamic properties [2]. Our findings build on Humphreys et al. [17], which linked the residency time of Blue-winged Teal (*Spatula discors*) at poultry houses to HPAIv occurrence, noting that dabbling ducks can have infection rates 10 times higher than geese [1].

We documented the first case of fine-scale goose movement near poultry facilities in the Mid-Atlantic, finding minimum distances of 3.9 m for CANG and 1.7 m for GSGO. Waterfowl can drive HPAIv introductions within poultry facilities [26,27]. In a study by McDuie et al. [7], 11 marked wild ducks of three species were tracked near commercial livestock facilities in California and Washington State. Similarly, the geese marked in this study utilized crop fields near poultry facilities during both day and night. We also analyzed the impact of farm clustering on wintering goose exposure; while it had no relationship with CANG exposure, it was significantly correlated with GSGO exposure when more than 40 farms were within a 2 km buffer. Farm clustering may be even more significant if the facility is within a control zone that is established once HPAIv is detected on a farm or production premises. Green et al. [26] have shown that this increases the risk of HPAIv introduction by 8 times. Large flocks of GSGO may be attracted to clustered farms to increase the likelihood of finding a reliable source of waste grain. The observation of a large flock (≥500,000) near farms was shown to increase introduction risk by 1.6 times [26]. Although difficult to quantify, an interesting question pertains to waterfowl memory and whether they return to the same areas near facilities for many years, particularly for long-lived species such as geese. With more GPS-marking of waterfowl species, we may begin to understand the extent to which areas near poultry farms are used consistently by waterfowl. For example, GPS-marking data demonstrated the return of a Mallard to a California livestock facility in the subsequent year [7].

Although the BRT models explained a modest proportion of deviance, 25.17 ± 2.65% (mean ± SE) and 16.68 ± 0.50% (mean ± SE) for CANG and GSGO, respectively, this level of explanatory power is not uncommon for telemetry-based studies of animal movement, where behavior is influenced by numerous factors operating across spatial and temporal scales [85–87]. Telemetry datasets inherently contain substantial variability among individuals and flocks, and much of this variation cannot be captured using landscape-scale environmental covariates alone. Unexplained variance may reflect factors such as individual behavioral differences [88,89], daily flock dynamics [90], temporary food availability [91], crop type and harvest status [92–94], waste grain abundance [30,92], hunting pressure/disturbance [36,95–97], fine-scale weather conditions [98], small water features not represented in national datasets [31,99], land management activities, and roost-site fidelity [100,101]. Despite this unexplained variation, the models consistently identified a small set of dominant predictors across cross-validation iterations, providing confidence in the ecological relevance of the highest-ranked variables (relative importance >1.0). Consequently, our conclusions are focused primarily on these strong and consistent predictors of winter habitat use, whereas interpretations of lower-ranked predictors should be viewed more cautiously due to their comparatively weak influence and greater sensitivity to unmeasured sources of variation.

GSGO exposure at poultry facilities peaks in mid-late winter when most of the population is present in the study area, with colder temperatures in January and February influencing their behavior and crop use. A minor increase in GSGO exposure in mid-late February may result from geese foraging near poultry facilities before migrating north [30,83,102,103]. CANG exposure remains consistent throughout winter, except for increased variability in late November to early December, coinciding with the start of Canada Goose hunting season, which affects their behavior due to hunting pressure [104–106]. Both species showed increasing exposure during colder temperatures, with CANG demonstrating a stronger relationship than GSGO. Only CANG also showed increasing exposure during warmer winter temperatures.

One explanation for increased CANG exposure with rising winter temperatures is that geese may shift from feeding in agricultural fields (near poultry facilities) to using open water or urban areas for thermoregulation (farther from poultry facilities) during colder periods [83]. These behaviors are not necessarily mutually exclusive and may depend on the geese’s familiarity with different habitats. The data showed that GSGO exposure was higher in colder temperatures, especially in areas with more developed land (Fig 4B). This suggests that geese may utilize urban spaces as thermal refugia and seek waste grain near poultry facilities. Our findings indicate that geese use open water as a thermoregulatory strategy below 0°C, but they tend to continue to forage in grain fields even in temperatures as low as −23.3°C [77,107]. In colder conditions, greater crop availability significantly increased GSGO exposure as geese offset thermoregulatory costs by foraging on energy-dense waste grain. The effects of temperature and land cover were more significant for CANG with increasing hay/pasture in cold winters and more grassland in warmer conditions, where warmer temperatures may enhance forage availability through ‘green up.’

Increasing temperatures lead to greater foraging flight distances for wintering dabbling ducks in Europe, likely due to lower thermoregulation costs [108]. Consequently, warmer temperatures may increase encounters with poultry farms. In contrast, Hill and Frederick [29] found that GSGO in the DP flew 368 m farther for each degree Celsius drop in temperature. Research (summarized in S3 Fig.) indicated that flight distances of GSGO and CANG increased below freezing, peaking at −20°C. Unlike dabbling ducks, larger-bodied wintering geese still fly in extreme cold, with GSGO flying at temperatures as low as –25°C. The caloric needs for thermoregulation drive wintering geese to make feeding flights to adjacent crop fields, where agricultural waste grain provides high caloric intake [30,77,83,109,110]. This pattern is also seen in the Mississippi Valley, where wintering CANG consume higher-energy foods as temperatures decline [77].

Geese typically roost at night in open water for thermoregulation and to avoid predators, but they may also feed during warmer nights [83,111–113]. This feeding allows them to replenish energy without the pressures of hunting [95]. In freezing temperatures, geese need to find high-calorie food to support thermoregulation, with waste grains such as corn and soybeans providing more energy than natural sources [77,109]. They may abandon a feeding area if it is no longer nutritionally sustainable [114]. While increasing temperatures did not affect GSGO exposure, they did lead to higher CANG exposure and allowed geese to expand their wintering ranges due to milder conditions and increased food availability [115,116].

Geographical features like sewage treatment facilities, waterbodies, and adjacent farms influenced goose exposure in winter. GSGO exposure at poultry facilities increased as distances decreased to the nearest waterbody offering roosting [77], thermoregulation [36,117], food, and/or refuge locations to geese. Waterbodies also heighten risks for AIV outbreaks in poultry [25,118,119]. In contrast, CANG exposure was positively related to distance from waterbodies, suggesting they prefer natural wetlands over larger sites used by GSGO, such as reservoirs, quarries, or wildlife refuges, which are all known to provide roosting areas [29,30,116]. A higher proportion of woody wetlands predicted CANG exposure, as they serve as roosting habitats. However, winter conditions may limit access to shallow wetlands, impacting exposure due to temperature. Landscapes with dominant woody wetlands and minimal open water (<15%) were ideal for CANG exposure, while increased woody wetlands reduced GSGO exposure.

Distance to sewage treatment facilities is significantly more predictive of GSGO and CANG exposure than distance to other features like waterbodies and coasts. These facilities serve as crucial habitats for waterfowl, particularly in areas where water is scarce [99], and for food resources [93,120,121]. Sewage facilities likely provide sanctuary during the hunting season and enable interactions with other birds, such as Red-winged Blackbirds (*Agelaius phoeniceus*) or other smaller passerine species, which could facilitate the spread of avian influenza (HPAIv) within poultry facilities [8,70]. Although sewage treatment facilities may offer some thermal benefits, their impact varied by species; GSGO exposure rises in colder weather, while CANG exposure increases along with temperature (Figs 5A and 6A). Additionally, these sites can harbor human and zoonotic diseases, and pathogens in waterfowl droppings may stem from human or domestic animal waste [2,33,122].

GSGO exposure was highest for facilities within 25 km of the coast, where many NWRs and wildlife management areas provide winter refuge for waterfowl before foraging. The average distance of DP poultry facilities (n = 6,021) from the coast was 24.83 ± 0.14 km and from NWRs was 19.96 ± 0.12 km. Previous studies indicate GSGO can fly 11–23 km from roost to feeding sites [29,110]. This study observed winter flight distances ranging from 0.31 to 138.13 km, suggesting birds roosted at coastal sites before foraging inland, where energy-rich waste grain may help geese travel further. In contrast, CANG exposure rates increased with distance from the coast, suggesting inland sites may be more significantly at risk from CANG. While CANG typically forage within 2.2 km [110], they are known to travel long distances in response to depleting resources in Wisconsin and Illinois [77]. CANG flights ranged from ~0–55 km during the winter in this study, but they flew comparatively far less on average than GSGO. CANG may make flights from coastal roosting sites to feed; however, the strength of the response between 41–44 km may indicate alternate inland roosting sites supporting wintering CANG.

Our findings indicate that National Wildlife Refuges (NWRs) play a crucial role in supporting wintering CANG [111] and GSGO [29] in the Delmarva Peninsula. The establishment of NWR sanctuaries that are closed to hunting and the availability of waste grain have contributed to population increases and improved survival rates of these geese [115]. CANG exposure was significantly higher near NWRs, highlighting their importance for resident CANG in winter. For GSGO, the varying patterns of exposure may be due to some geese roosting in coastal NWRs, while others choose alternative inland sites. Based on observations by our team and others [29], large flocks of GSGO often roost in NWRs before feeding in nearby agricultural fields or within coastal *Spartina spp.* wetlands. Their usage of NWRs likely fluctuates with resource availability [123], particularly corn, which is more abundant earlier in winter before depletion and crop preparation begins [29]. Increased exposure of facilities by both goose species was noted within 2 km of NWRs, suggesting that facilities in proximity to Refuges experience careful site management considerations.

Waterbodies near poultry farms can serve as refuges for geese, providing a place to roost before feeding in adjacent farmlands. Our study found that the presence of wintering geese was influenced by both the distance and type of waterbody. When within facility buffers (2 km from facilities), CANG were primarily observed near rivers and streams, while GSGO preferred swamps/marshes. The relationship between distance to waterbody and goose exposure was consistent across the winter period. Further investigation into the type and size of waterbodies would help elucidate their impact on the presence of wintering waterfowl.

Our research indicates that while both CANG and GSGO utilize farm ponds near poultry facilities, these ponds were not significantly linked to their presence. Other research indicated that agricultural wastewater lagoons and drainage ditches within 320 m of a facility increased the HPAIv introduction risk by an average of 3.3 times, as they provide feeding and roosting sites [26]. Biosecurity measures are essential when managing farm ponds near poultry operations, especially because geese frequent these areas, and particularly in agricultural regions that grow feed for poultry and livestock [7,31]. Open water bodies can serve as reservoirs for AIV, increasing infection risks [14,22,25,124]. Airborne pathways for AIV entry, especially within 100 m, are also factored in [11,125]. Current efforts to manage HPAIv in facilities involve significant human activity that may aerosolize infected materials without sufficient awareness of air movement. To inform this issue, future research could examine how the size and depth of surface water bodies and buffer zones affect wintering waterfowl exposure.

Upland habitats significantly influenced the exposure of both goose species at poultry facilities, with GSGO showing a stronger positive association with crop availability. GSGO responses to crops fluctuate during winter based on waste grain resources and temperature. Our estimates suggest that the highest GSGO exposure often occurs in agricultural fields with low forest cover (<1%) and around 40% developed land. Interestingly, barren land cover was unexpectedly associated with high CANG exposure, whereas GSGO had a weaker response. Barren patches, like quarries, can serve as refuge sites for CANG. As expected, since grasses are a food source for geese [23,29,126], CANG exposure increased with both hay/pasture and grasslands. GSGO also increased with hay/pasture, indicating it may attract wintering GSGO during certain times despite a lower magnitude response than CANG.

Our results indicate that GSGO and CANG exposure rates were similar during day and night, suggesting that relying solely on daytime surveillance may not adequately assess 24-hour poultry facility exposure to waterfowl. While nighttime goose presence may typically indicate roosting sites near farms, studies show that geese can shift their feeding to nocturnal periods due to energetic demands or disturbances like hunting [36,104,127,128]. For GSGO, exposure significantly increased—by about four to six times—when the crop proportion within facility buffers exceeded 70%, indicating that adjacent waste grain is a strong attractant. In contrast, CANG exposure was less influenced by crop proportions and was affected more by factors like the week of the year and proximity to features such as sewage treatment facilities, NWRs, and water bodies.

Areas of high waterfowl use and poultry facility presence create infection pathways via the accumulation of excrement and subsequent run-off, which may enter facilities or nearby waterbodies. Poultry that use open waters are known to become infected by wild waterfowl that have previously congregated in the area [28]. The accumulation of excrement from large aggregations of waterfowl at farms also creates pathways for human (foot and vehicle traffic) entry of HPAIv into poultry facilities, especially if workers are moving between facilities or proper biosecurity is not being practiced [26]. Waterfowl in proximity to poultry facilities also have the capacity to interface with other potential HPAIv reservoirs that are commonly associated with human habitation, such as House Sparrows (*Passer domesticus*), European Starlings (*Sturnus vulgaris*), Rock Pigeons (*Columba livia*), gulls (*Laridae spp.*), and crows (*Corvidae spp*.) [129–131], which could lead to HPAIv spillover into poultry.

To demonstrate the effectiveness of the percentile risk ranking system for predicting HPAIv outbreaks at facilities, we retrospectively assessed whether these facilities could have been flagged prior to the H5N1 outbreak based on cumulative exposure since the first wild bird detection in the USA. All seven facilities infected with HPAIv in the DP were ranked within the top 25% priority farms, with one infected facility being one of the top-25 facilities flagged based on risk (i.e., top 0.4%). Our methodology for classifying facilities into the top 25% most exposed (~1,500 facilities) could be a criteria for focusing biosecurity efforts when resources of response teams (i.e., veterinarians) are limited.

To ensure biosecurity at poultry facilities beyond standard biosecurity measures, managers could focus on disease risk management throughout winter, particularly when temperatures are below freezing and wintering geese, especially GSGO, are more likely to visit facilities in search of food, such as waste grain. Clustered facilities, those within 5 km of key waterfowl concentration areas (e.g., sewage treatment plants, wildlife refuges, and water bodies) and those within 20 km of the Atlantic coast, face higher exposure to geese and should be particularly vigilant. Additionally, inland facilities near large water sources within 5 km could benefit from monitoring geese more closely, as these areas serve as important roosting spots before foraging.

Our research, alongside other studies [7,17], has quantified goose activity near poultry production facilities. Increased bird interactions raise the risk of HPAIv outbreaks [26,27]. While our primary goal was to identify environmental factors that increase the likelihood of waterfowl-poultry interactions, the importance of effective biosecurity measures is crucial. Wild birds may be drawn to poultry farms, but human activity often bridges the gap between environmental viruses and susceptible hosts. Risky practices in poultry production, which enable animal-wild bird interactions, are still accepted by the industry [132,133]. Despite the large number of waterfowl-farm interactions, outbreak incidence remains relatively low. Our biologging studies aid in understanding how the environment influences bird movement and land use, which is vital as HPAIv becomes endemic in North America. Biologging data enables farmers to make informed decisions based on bird activity and outbreak risks. Additionally, this data can be used to help evaluate and select potential refuge spaces for wild birds, particularly in areas with high poultry production or risk management needs. Future research could explore risk rankings based on cumulative exposure days across multiple buffer distances and evaluate their ability to predict historical HPAIv outbreaks, similar to the approach presented here using 2-km buffers.

## Supporting information

S1 Table Capture locations and year for 2 species of geese marked with GPS transmitters during 2019–2021. (TIF)

S1 Fig Poultry facility density and telemetry locations of Greater Snow Geese and Canada Geese on the Delmarva Peninsula. Map of A) number of poultry farms (Soroka and Duren 2020) within 2 km resolution grid cells within the Delmarva Peninsula, with H5N1 infected counties (N = 4) highlighted with yellow, B) total telemetry locations for individual Greater Snow Goose (N = 59) during winters 2019–2023, and C) total telemetry locations for individual Canada Goose (N = 9) during winters 2021–2023. U.S. Census Bureau TIGER/Line administrative boundaries.
(TIF)

S2 Fig Effects of lower-importance predictors on Greater Snow Goose and Canada Goose exposure at poultry facilities. Mean relative Greater Snow Goose (top A-H) and Canada Goose (bottom A-H) points per hour at Delmarva Peninsula poultry facilities as a function of predictors with mean relative response values <1.0 across 25 Boosted Regression Tree models across 4 winters (1 November – 15 March 2019–2023). Shading represents SE of mean response among 25 Boosted Regression Tree models. Error bars (whiskers) indicate ±1 SE.
(TIF)

S3 Fig Temperature and Flight Activity of Wintering Geese. Interactive effects of average temperature and proportion of time spent in flight across 4 winters (1 November – 15 March 2019–2023 for Greater Snow Goose (A) and Canada Goose (B) in the Delmarva Peninsula. Shaded area represents standard error.
(TIF)

## References

[1] Olsen B, Munster VJ, Wallensten A, Waldenström J, Osterhaus ADME, Fouchier RAM. Global patterns of influenza a virus in wild birds. Science. 2006;312(5772):384–8. doi: 10.1126/science.1122438 16627734

[2] Elmberg J, Berg C, Lerner H, Waldenström J, Hessel R. Potential disease transmission from wild geese and swans to livestock, poultry and humans: a review of the scientific literature from a One Health perspective. Infect Ecol Epidemiol. 2017;7(1):1300450. doi: 10.1080/20008686.2017.1300450 28567210 PMC5443079

[3] Perkins LE, Swayne DE. Pathobiology of A/Chicken/Hong Kong/220/97 (H5N1) avian influenza virus in seven gallinaceous species. Vet Pathol. 2001;38:149–64. doi: 10.1354/vp.38-2-149 11280371

[4] Swayne DE. Avian influenza. 1st ed. Ames, Iowa: Blackwell. 2008. http://catdir.loc.gov/catdir/enhancements/fy0805/2007039629-t.html

[5] Acosta S, Kelman T, Feirer S, Matchett E, Smolinsky J, Pitesky M, et al. Using the California Waterfowl Tracker to Assess Proximity of Waterfowl to Commercial Poultry in the Central Valley of California. Avian Dis. 2021;65(3):483–92. doi: 10.1637/aviandiseases-D-20-00137 34699147

[6] Madsen JM, Zimmermann NG, Timmons J, Tablante NL. Avian influenza seroprevalence and biosecurity risk factors in Maryland backyard poultry: a cross-sectional study. PLoS One. 2013;8(2):e56851. doi: 10.1371/journal.pone.0056851 23437257 PMC3577693

[7] McDuie F, Matchett EL, Prosser DJ, Takekawa JY, Pitesky ME, Lorenz AA, et al. Pathways for avian influenza virus spread: GPS reveals wild waterfowl in commercial livestock facilities and connectivity with the natural wetland landscape. Transbound Emerg Dis. 2022;69(5):2898–912. doi: 10.1111/tbed.14445 34974641 PMC9788224

[8] USDA-APHIS. Confirmed pathogenic avian flu in commercial & backyard flocks. APHIS. https://www.aphis.usda.gov/livestock-poultry-disease/avian/avian-influenza/hpai-detections/commercial-backyard-flocks. Accessed 2026 June 16.

[9] Lebarbenchon C, Feare CJ, Renaud F, Thomas F, Gauthier-Clerc M. Persistence of highly pathogenic avian influenza viruses in natural ecosystems. Emerg Infect Dis. 2010;16(7):1057–62. doi: 10.3201/eid1607.090389 20587174 PMC3321889

[10] Lang AS, Kelly A, Runstadler JA. Prevalence and diversity of avian influenza viruses in environmental reservoirs. J Gen Virol. 2008;89(Pt 2):509–19. doi: 10.1099/vir.0.83369-0 18198382

[11] Zhang JL, Chen Z-Y, Lin S-L, King C-C, Chen C-C, Chen P-S. Airborne avian influenza virus in ambient air in the winter habitats of migratory birds. Environ Sci Technol. 2022;56(22):15365–76. doi: 10.1021/acs.est.2c04528 36288568

[12] Krauss S, Webster RG. Avian influenza virus surveillance and wild birds: past and present. Avian Dis. 2010;54(1 Suppl):394–8. doi: 10.1637/8703-031609-Review.1 20521668

[13] Ramey AM, Reeves AB, Lagassé BJ, Patil V, Hubbard LE, Kolpin DW, et al. Evidence for interannual persistence of infectious influenza A viruses in Alaska wetlands. Sci Total Environ. 2022;803:150078. doi: 10.1016/j.scitotenv.2021.150078 34525758 PMC9277558

[14] Brown JD, Goekjian G, Poulson R, Valeika S, Stallknecht DE. Avian influenza virus in water: infectivity is dependent on pH, salinity and temperature. Vet Microbiol. 2009;136(1–2):20–6. doi: 10.1016/j.vetmic.2008.10.027 19081209

[15] Spackman E. A brief introduction to avian influenza virus. Methods Mol Biol. 2014;1161:61–8. doi: 10.1007/978-1-4939-0758-8_6 24899420

[16] Densmore CL, Iwanowicz DD, McLaughlin SM, Ottinger CA, Spires JE, Iwanowicz LR. Influenza A virus detected in native bivalves in waterfowl habitat of the Delmarva peninsula, USA. Microorganisms. 2019;7(9):334. doi: 10.3390/microorganisms7090334 31505778 PMC6780145

[17] Humphreys JM, Douglas DC, Ramey AM, Mullinax JM, Soos C, Link P, et al. The spatial–temporal relationship of blue‐winged teal to domestic poultry: Movement state modelling of a highly mobile avian influenza host. J Appl Ecol. 2021;58:2040–52. doi: 10.1111/1365-2664.13963

[18] Girard YA, Runstadler JA, Aldehoff F, Boyce W. Genetic structure of Pacific Flyway avian influenza viruses is shaped by geographic location, host species, and sampling period. Virus Genes. 2012;44(3):415–28. doi: 10.1007/s11262-011-0706-5 22222690

[19] Gass JD, Hill NJ, Damodaran L, Naumova EN, Nutter FB, Runstadler JA. Ecogeographic drivers of the spatial spread of highly pathogenic avian influenza outbreaks in Europe and the United States, 2016-early 2022. Int J Environ Res Public Health. 2023;20(11):6030. doi: 10.3390/ijerph20116030 37297634 PMC10252585

[20] Cappelle J, Gaidet N, Newman S, Takekawa JY, Prosser DJ, Iverson S, et al. Potential dispersal range and rate of H5N1 HPAI virus by wild waterfowl: estimation from satellite-tracked bird movements. In: 7th Conference of the European Ornithologists Union 21-26 August 2009, Zurich, Switzerland: abstracts [Internet]. Swiss Ornithological Institute; 2009 [cited 31 Jan 2023]. https://agritrop.cirad.fr/573585/

[21] Fouchier R, Munster V. Epidemiology of low pathogenic avian influenza viruses in wild birds. Rev Sci Tech OIE. 2009;28:49–58. doi: 10.20506/rst.28.1.1863 19618618

[22] Humphreys JM, Ramey AM, Douglas DC, Mullinax JM, Soos C, Link P, et al. Waterfowl occurrence and residence time as indicators of H5 and H7 avian influenza in North American Poultry. Sci Rep. 2020;10(1):2592. doi: 10.1038/s41598-020-59077-1 32054908 PMC7018751

[23] Buij R, Melman TCP, Loonen MJJE, Fox AD. Balancing ecosystem function, services and disservices resulting from expanding goose populations. Ambio. 2017;46(Suppl 2):301–18. doi: 10.1007/s13280-017-0902-1 28215006 PMC5316333

[24] Lee K, Yu D, Martínez-López B, Yoon H, Kang S-I, Hong S-K, et al. Fine-scale tracking of wild waterfowl and their impact on highly pathogenic avian influenza outbreaks in the Republic of Korea, 2014-2015. Sci Rep. 2020;10(1):18631. doi: 10.1038/s41598-020-75698-y 33122803 PMC7596240

[25] Ahmad S, Koh K, Yoo D, Suh G, Lee J, Lee C-M. Impact of inland waters on highly pathogenic avian influenza outbreaks in neighboring poultry farms in South Korea. J Vet Sci. 2022;23(3):e36. doi: 10.4142/jvs.21278 35618317 PMC9149499

[26] Green AL, Branan M, Fields VL, Patyk K, Kolar SK, Beam A, et al. Investigation of risk factors for introduction of highly pathogenic avian influenza H5N1 virus onto table egg farms in the United States, 2022: a case-control study. Front Vet Sci. 2023;10:1229008. doi: 10.3389/fvets.2023.1229008 37559891 PMC10408129

[27] Patyk KA, Fields VL, Beam AL, Branan MA, McGuigan RE, Green A, et al. Investigation of risk factors for introduction of highly pathogenic avian influenza H5N1 infection among commercial turkey operations in the United States, 2022: a case-control study. Front Vet Sci. 2023;10:1229071. doi: 10.3389/fvets.2023.1229071 37711433 PMC10498466

[28] Cappelle J, Gaidet N, Iverson SA, Takekawa JY, Newman SH, Fofana B, et al. Characterizing the interface between wild ducks and poultry to evaluate the potential of transmission of avian pathogens. Int J Health Geogr. 2011;10:60. doi: 10.1186/1476-072X-10-60 22085837 PMC3280937

[29] Hill MRJ, Frederick RB. Winter movements and habitat use by greater snow geese. The Journal of Wildlife Management. 1997;61(4):1213. doi: 10.2307/3802119

[30] Jefferies RL, Rockwell RF, Abraham KF. The embarrassment of riches: agricultural food subsidies, high goose numbers, and loss of Arctic wetlands — a continuing saga. 2003;11:41.

[31] Sullivan JD, McDonough AM, Lescure LM, Prosser DJ. Identifying an understudied interface: preliminary evaluation of the use of retention ponds on commercial poultry farms by wild waterfowl. Transboundary and Emerging Diseases. 2024;2024:3022927. doi: 10.1155/2024/3022927 40303142 PMC12016893

[32] Lazur A, Hengst A, Markin E, Webster D, Billing K, Schuck D. Urban and stormwater pond management.

[33] Lu J, Santo Domingo JW, Hill S, Edge TA. Microbial diversity and host-specific sequences of Canada goose feces. Appl Environ Microbiol. 2009;75(18):5919–26. doi: 10.1128/AEM.00462-09 19633110 PMC2747875

[34] Mott AL. Habitat use and distribution implications of four goose species wintering in California’s Sacramento Valley. 2023.

[35] Schreven KHT, Stolz C, Madsen J, Nolet BA. Nesting attempts and success of Arctic-breeding geese can be derived with high precision from accelerometry and GPS-tracking. Anim Biotelemetry. 2021;9(1). doi: 10.1186/s40317-021-00249-9

[36] Askren RJ, Eichholz MW, Sharp CM, Washburn BE, Beckerman SF, Pullins CK, et al. Behavioral responses of Canada geese to winter harassment in the context of human‐wildlife conflicts. Wildlife Society Bulletin. 2022;46(5). doi: 10.1002/wsb.1384

[37] Trimbake P. Sleep-swimming in Canada geese (Branta canadensis). 2023. http://dr.iiserpune.ac.in:8080/xmlui/handle/123456789/7949

[38] Kölzsch A, Lameris TK, Müskens GJDM, Schreven KHT, Buitendijk NH, Kruckenberg H, et al. Wild goose chase: Geese flee high and far, and with aftereffects from New Year’s fireworks. Conservation Letters. 2022;16(1). doi: 10.1111/conl.12927

[39] Yamaguchi N, Hiraoka E, Fujita M, Hijikata N, Ueta M, Takagi K, et al. Spring migration routes of mallards (Anas platyrhynchos) that winter in Japan, determined from satellite telemetry. Zoolog Sci. 2008;25(9):875–81. doi: 10.2108/zsj.25.875 19267595

[40] Takekawa JY, Newman SH, Xiao X, Prosser DJ, Spragens KA, Palm EC. Migration of waterfowl in the East Asian flyway and spatial relationship to HPAI H5N1 outbreaks. 2023. 10.1637/8914-043009-Reg.1 PMC4878034 20521681

[41] National Audubon Society. Important Bird Areas | Audubon. https://www.audubon.org/important-bird-areas. Accessed 2026 June 16.

[42] Delaware Climate Office. DCO Home Page. Delaware Climate Office. https://climate.udel.edu/. Accessed 2026 June 16.

[43] National Oceanic and Atmospheric Administration. Climate. https://www.noaa.gov/climate. Accessed 2026 June 16.

[44] Soroka A, Duren Z. Poultry Feeding Operations on the Delaware, Maryland, and Virginia Peninsula from 2016 to 2017. U.S. Geological Survey. https://www.usgs.gov/data/poultry-feeding-operations-delaware-maryland-and-virginia-peninsula-2016-2017. Accessed 2026 June 16.

[45] U.S. Department of Agriculture, National Agricultural Statistics Service (USDA NASS). List of Reports and Publications | 2022 Census of Agriculture | USDA/NASS. https://www.nass.usda.gov/Publications/AgCensus/2022/?utm_source=chatgpt.com#full_report. Accessed 2026 July 1.

[46] Dahl T. Wetland losses in the United States, 1780s to 1980s. U.S. Fish and Wildlife Service. 1990. https://www.fws.gov/media/wetland-losses-united-states-1780s-1980s

[47] Evrard JO, Bacon BR. Duck trapping success and mortality using four trap designs. doi: N/A

[48] Morez V, Gauthier G, Reed A. Effect of body condition on vulnerability of greater snow geese to hunting and capture. The Journal of Wildlife Management. 2000;64(3):875. doi: 10.2307/3802758

[49] Kays R, Davidson SC, Berger M, Bohrer G, Fiedler W, Flack A, et al. The Movebank system for studying global animal movement and demography. Methods Ecol Evol. 2021;13(2):419–31. doi: 10.1111/2041-210x.13767

[50] Kranstauber B, Smolla M, Scharf AK. Move: visualizing and analyzing animal track data. 2018.

[51] R Development Core Team. R: The R Project for Statistical Computing. https://www.r-project.org/. Accessed 2026 June 16.

[52] Delmarva Chicken Association USP& E. Welcome to the Delmarva Chicken Association Web Site. https://www.dcachicken.com/. Accessed 2026 June 16.

[53] Signer J, Fieberg J, Avgar T. Animal movement tools (amt): R package for managing tracking data and conducting habitat selection analyses. Ecol Evol. 2019;9(2):880–90. doi: 10.1002/ece3.4823 30766677 PMC6362447

[54] Michelot T, Langrock R, Patterson TA. moveHMM: an R package for the statistical modelling of animal movement data using hidden Markov models. Methods Ecol Evol. 2016;7(11):1308–15. doi: 10.1111/2041-210x.12578

[55] McDuie F, Casazza ML, Keiter D, Overton CT, Herzog MP, Feldheim CL. Moving at the speed of flight: dabbling duck-movement rates and the relationship with electronic tracking interval. Wildlife Research. 2019;46:533–43. doi: 10.1071/WR19028

[56] McDuie F, Overton C, Lorenz A, Matchett E, Mott A, Mackell D, et al. Mitigating risk: predicting H5N1 avian influenza spread with an empirical model of bird movement. Transboundary and Emerging Diseases. 2024;2024:5525298. doi: 10.1155/2024/5525298 40303041 PMC12016750

[57] Soroka AM, Rosenbloom NW, King AM, Li W, Dinh A, Petenbrink MN. Water bodies within 500 meters of poultry feeding operations on the Delmarva Peninsula in 2016 and 2017. U.S. Geological Survey. 2020. doi: 10.5066/P9PSQ8IP

[58] USGS. National Hydrography Dataset. U.S. Geological Survey. https://www.usgs.gov/national-hydrography/national-hydrography-dataset. Accessed 2026 June 16.

[59] Ehalt Macedo H, Lehner B, Nicell J, Grill G, Li J, Limtong A, et al. Distribution and characteristics of wastewater treatment plants within the global river network. Earth Syst Sci Data. 2022;14(2):559–77. doi: 10.5194/essd-14-559-2022

[60] U.S. Geological Survey. Protected Areas. https://www.usgs.gov/programs/gap-analysis-project/science/protected-areas. 2022. Accessed 2026 June 16.

[61] Hersbach H, Bell B, Berrisford P, Hirahara S, Horányi A, Muñoz-Sabater J. The ERA5 global reanalysis. 2026. doi: 10.1002/qj.3803

[62] Dewitz J. National Land Cover Database (NLCD) 2019 Products (ver. 3.0, February 2024). U.S. Geological Survey. 2024. doi: 10.5066/P9KZCM54

[63] Thieurmel B. suncalc. DataStorm; 2023. https://github.com/datastorm-open/suncalc

[64] Hijmans R. dismo. rspatial. https://github.com/rspatial/dismo. 2023.

[65] Elith J, Leathwick JR, Hastie T. A working guide to boosted regression trees. J Anim Ecol. 2008;77(4):802–13. doi: 10.1111/j.1365-2656.2008.01390.x 18397250

[66] Robinson WS. Ecological correlations and the behavior of individuals. American Sociological Review. 1950;15(3):351. doi: 10.2307/2087176

[67] Cramer JS. Efficient grouping, regression and correlation in engel curve analysis. Journal of the American Statistical Association. 1964;59(305):233–50. doi: 10.1080/01621459.1964.10480714

[68] Cam E, Link WA, Cooch EG, Monnat J-Y, Danchin E. Individual covariation in life-history traits: seeing the trees despite the forest. Am Nat. 2002;159(1):96–105. doi: 10.1086/324126 18707403

[69] Clark JS. Uncertainty and variability in demography and population growth: a hierarchical approach. Ecology. 2003;84(6):1370–81. doi: 10.1890/0012-9658(2003)084[1370:uavida]2.0.co;2

[70] USDA-APHIS. HPAI Detections in Wild Birds. https://www.aphis.usda.gov/livestock-poultry-disease/avian/avian-influenza/hpai-detections/wild-birds. Accessed 2026 June 22.

[71] Canadian Food Inspection Agency. National avian influenza - wild positives. High pathogenicity avian influenza in wildlife. https://www.arcgis.com/apps/dashboards/89c779e98cdf492c899df23e1c38fdbc. Accessed 2026 June 22.

[72] Prosser DJ, Takekawa JY, Newman SH, Yan B, Douglas DC, Hou Y, et al. Satellite‐marked waterfowl reveal migratory connection between H5N1 outbreak areas in China and Mongolia. Ibis. 2009;151(3):568–76. doi: 10.1111/j.1474-919x.2009.00932.x

[73] Gaidet N, Cappelle J, Takekawa JY, Prosser DJ, Iverson SA, Douglas DC, et al. Potential spread of highly pathogenic avian influenza H5N1 by wildfowl: dispersal ranges and rates determined from large‐scale satellite telemetry. Journal of Applied Ecology. 2010;47(5):1147–57. doi: 10.1111/j.1365-2664.2010.01845.x

[74] U.S. Fish & Wildlife Service. Waterfowl Population Status Reports. https://www.fws.gov/library/collections/waterfowl-population-status-reports. Accessed 2026 June 16.

[75] Smith TJ, Odum WE. The effects of grazing by snow geese on coastal salt marshes. Ecology. 1981;62(1):98–106. doi: 10.2307/1936673

[76] Reese JG, Weterings R. Waterfowl migration chronologies in central Chesapeake Bay during 2002–2013. The Wilson Journal of Ornithology. 2018;130(1):52–69. doi: 10.1676/16-043.1

[77] Gates RJ, Caithamer DF, Moritz WE, Tacha TC. Bioenergetics and nutrition of Mississippi Valley population Canada geese during winter and migration. 2020;68.

[78] Brown JD, Stallknecht DE, Beck JR, Suarez DL, Swayne DE. Susceptibility of North American ducks and gulls to H5N1 highly pathogenic avian influenza viruses. Emerg Infect Dis. 2006;12(11):1663–70. doi: 10.3201/eid1211.060652 17283615 PMC3372354

[79] Groepper SR, DeLiberto TJ, Vrtiska MP, Pedersen K, Swafford SR, Hygnstrom SE. Avian influenza virus prevalence in migratory waterfowl in the United States, 2007-2009. Avian Dis. 2014;58:531–40. doi: 10.1637/10849-042214-Reg.1 25618997

[80] Samuel MD, Hall JS, Brown JD, Goldberg DR, Ip H, Baranyuk VV. The dynamics of avian influenza in Lesser Snow Geese: implications for annual and migratory infection patterns. Ecol Appl. 2015;25(7):1851–9. doi: 10.1890/14-1820.1 26591451

[81] Ward MP, Maftei DN, Apostu CL, Suru AR. Association between outbreaks of highly pathogenic avian influenza subtype H5N1 and migratory waterfowl (family Anatidae) populations. Zoonoses Public Health. 2009;56(1):1–9. doi: 10.1111/j.1863-2378.2008.01150.x 18793277

[82] Cappelle J, Girard O, Fofana B, Gaidet N, Gilbert M. Ecological modeling of the spatial distribution of wild waterbirds to identify the main areas where avian influenza viruses are circulating in the Inner Niger Delta, Mali. Ecohealth. 2010;7(3):283–93. doi: 10.1007/s10393-010-0347-5 20865438

[83] Dorak BE, Ward MP, Eichholz MW, Washburn BE, Lyons TP, Hagy HM. Survival and habitat selection of Canada Geese during autumn and winter in metropolitan Chicago, USA. Condor. 2017;119:787–99. doi: 10.1650/CONDOR-16-234.1

[84] Kear J. The agricultural importance of wild goose droppings. Wildfowl. 1963;14:72–7.

[85] Hertel AG, Niemelä PT, Dingemanse NJ, Mueller T. A guide for studying among-individual behavioral variation from movement data in the wild. Mov Ecol. 2020;8:30. doi: 10.1186/s40462-020-00216-8 32612837 PMC7325061

[86] Gurarie E, Ovaskainen O. Characteristic spatial and temporal scales unify models of animal movement. Am Nat. 2011;178(1):113–23. doi: 10.1086/660285 21670582

[87] Nathan R, Getz WM, Revilla E, Holyoak M, Kadmon R, Saltz D, et al. A movement ecology paradigm for unifying organismal movement research. Proc Natl Acad Sci U S A. 2008;105(49):19052–9. doi: 10.1073/pnas.0800375105 19060196 PMC2614714

[88] Clobert J, Le Galliard J-F, Cote J, Meylan S, Massot M. Informed dispersal, heterogeneity in animal dispersal syndromes and the dynamics of spatially structured populations. Ecol Lett. 2009;12(3):197–209. doi: 10.1111/j.1461-0248.2008.01267.x 19170731

[89] Newton I. Learning and social influence on bird migration. Ardea. 2024;112(2). doi: 10.5253/arde.2024.a8

[90] Vickers SH, Meehan TD, Michel NL, Franco AMA, Gilroy JJ. North American avian species that migrate in flocks show greater long-term non-breeding range shift rates. Mov Ecol. 2025;13(1):3. doi: 10.1186/s40462-024-00527-0 39806506 PMC11730467

[91] Ely CR, Raveling DG. Seasonal variation in nutritional characteristics of the diet of greater white‐fronted geese. J Wildl Manag. 2011;75(1):78–91. doi: 10.1002/jwmg.13

[92] Hardy MJ, Williams CK, Ladman BS, Pitesky ME, Overton CT, Casazza ML, et al. Examining inter‐regional and intra‐seasonal differences in wintering waterfowl landscape associations among Pacific and Atlantic flyways. Journal of Avian Biology. 2024;2025(3). doi: 10.1111/jav.03296

[93] Elphick CS. Functional equivalency between rice fields and seminatural wetland habitats. Conservation Biology. 2000;14(1):181–91. doi: 10.1046/j.1523-1739.2000.98314.x

[94] Elphick CS, Oring LW. Conservation implications of flooding rice fields on winter waterbird communities. Agriculture, Ecosystems & Environment. 2003;94(1):17–29. doi: 10.1016/s0167-8809(02)00022-1

[95] Bélanger L, Bédard J. Responses of staging greater snow geese to human disturbance. The Journal of Wildlife Management. 1989;53:713–9. doi: 10.2307/3809202

[96] Beatty KE, Huck NR, Buderman FE. Anthropogenic predation risk alters waterfowl habitat selection. Landscape Ecology. 2024;39:201. doi: 10.1007/s10980-024-01995-w

[97] Masto NM, Blake-Bradshaw AG, Highway CJ, Keever AC, Feddersen JC, Hagy HM. Human access constrains optimal foraging and habitat availability in an avian generalist. Ecological Applications. n/a:e2952. doi: 10.1002/eap.2952 38417451

[98] O’Neal BJ, Stafford JD, Larkin RP, Michel ES. The effect of weather on the decision to migrate from stopover sites by autumn-migrating ducks. Mov Ecol. 2018;6:23. doi: 10.1186/s40462-018-0141-5 30505448 PMC6257954

[99] Dean WRJ, Milton SJ, Forsyth HP, Tissiman DR. Fluctuations in bird numbers on sewage treatment ponds in an arid environment, South Africa. Ostrich. 2015;86(1–2):145–53. doi: 10.2989/00306525.2015.1029559

[100] Wilson JC, Wood KA, Griffin LR, Brides K, Rees EC, Ezard THG. Using satellite tracking to assess the use of protected areas and alternative roosts by Whooper and Bewick’s Swans. Ibis. 2024;167(2):515–29. doi: 10.1111/ibi.13369

[101] Clausen KK, Clausen P, Hounisen JP, Vissing MS, Fox AD. Foraging range, habitat use and minimum flight distances of East Atlantic Light-bellied Brent Geese Branta bernicla hrota in their spring staging areas. 2013;15.

[102] Fan Y, Zhou L, Cheng L, Song Y, Xu W. Foraging behavior of the Greater White-fronted Goose (Anser albifrons) wintering at Shengjin Lake: diet shifts and habitat use. Avian Res. 2020;11(1). doi: 10.1186/s40657-020-0189-y

[103] Fowler DN, Webb EB, Vrtiska MP, Hobson KA. Winter carry-over effects on spring body condition driven by agricultural subsidies to Lesser Snow Geese (Anser caerulescens caerulescens). ACE. 2020;15(2). doi: 10.5751/ace-01743-150221

[104] Madsen J, Fox AD. Impacts of hunting disturbance on waterbirds ‐ a review. Wildlife Biology. 1995;1(4):193–207. doi: 10.2981/wlb.1995.0025

[105] Dalermoen RAR. Goose response to hunting activities.

[106] Leblanc ML, Hanson A, Leblon B, LaRocque A, Humphries MM. Hunting and seagrass affect fall stopover Canada goose distribution in eastern Canada. The Journal of Wildlife Management. 2023;(n/a):e22428. doi: 10.1002/jwmg.22428

[107] Davis SE, Klaas EE, Koehler KJ. Diurnal time-activity budgets and habitat use of Lesser Snow Geese Anser caerulescens in the middle Missouri River valley during winter and spring. Wildfowl. 1989;40:45–54.

[108] Legagneux P, Blaize C, Latraube F, Gautier J, Bretagnolle V. Variation in home-range size and movements of wintering dabbling ducks. J Ornithol. 2009;150:183–93. doi: 10.1007/s10336-008-0333-7

[109] Fredrickson LH, Reid FA. Nutritional values of waterfowl foods. 1988.

[110] Johnson WP, Schmidt PM, Taylor DP. Foraging flight distances of wintering ducks and geese: a review. ACE. 2014;9:art2. doi: 10.5751/ACE-00683-090202

[111] Raveling DG, Crews WE, Klimstra WD. Activity patterns of Canada Geese during winter. The Wilson Bulletin. 1972;84:18.

[112] Dorak BE, Hagy HM, Ward MP. Micro-site climatic conditions associated with urban thermal refugia used by Canada geese during winter. 2016. http://www.northamericanducksymposium.org/docs/NADS7Program/NADS7%20Program%20All%20Days%20All%20Abstracts.pdf

[113] Lameris TK, Dokter AM, van der Jeugd HP, Bouten W, Koster J, Sand SHH, et al. Nocturnal foraging lifts time constraints in winter for migratory geese but hardly speeds up fueling. Behav Ecol. 2021;32(3):539–52. doi: 10.1093/beheco/araa152 34104110 PMC8177807

[114] van Gils JA, Tijsen W. Short-term foraging costs and long-term fueling rates in central-place foraging swans revealed by giving-up exploitation times. Am Nat. 2007;169(5):609–20. doi: 10.1086/513114 17427132

[115] Gauthier G, Giroux J, Reed A, Béchet A, Bélanger L. Interactions between land use, habitat use, and population increase in greater snow geese: what are the consequences for natural wetlands?. Global Change Biology. 2005;11(6):856–68. doi: 10.1111/j.1365-2486.2005.00944.x

[116] Lefebvre J, Gauthier G, Giroux J-F, Reed A, Reed ET, Bélanger L. The greater snow goose Anser caerulescens atlanticus: Managing an overabundant population. Ambio. 2017;46(Suppl 2):262–74. doi: 10.1007/s13280-016-0887-1 28215008 PMC5316327

[117] Askren RJ, Massey ER, James JD, Osborne DC. Migration chronology and multi-scale habitat selection of wintering midcontinent greater white-fronted geese. Global Ecology and Conservation. 2022;39:e02290. doi: 10.1016/j.gecco.2022.e02290

[118] Iglesias I, Jesús Muñoz M, Martínez M, de la Torre A. Environmental risk factors associated with H5N1 HPAI in Ramsar wetlands of Europe. Avian Dis. 2010;54(2):814–20. doi: 10.1637/8970-062609-Reg.1 20608524

[119] Pérez-Ramírez E, Acevedo P, Allepuz A, Gerrikagoitia X, Alba A, Busquets N, et al. Ecological factors driving avian influenza virus dynamics in Spanish wetland ecosystems. PLoS One. 2012;7(11):e46418. doi: 10.1371/journal.pone.0046418 23152749 PMC3495955

[120] Andersen DC, Sartoris JJ, Thullen JS, Reusch PG. The effects of bird use on nutrient removal in a constructed wastewater-treatment wetland. Wetlands. 2003;23(2):423–35. doi: 10.1672/17-20

[121] Loyn R, Rogers D, Swindley RJ, Stamation K, Macak P, Menkhorst P. Waterbird monitoring at the Western Treatment Plant, 2000-12: The effects of climate and sewage treatment processes on waterbird populations. 2014. doi: 10.13140/RG.2.1.3428.1045

[122] Benskin CMH, Wilson K, Jones K, Hartley IR. Bacterial pathogens in wild birds: a review of the frequency and effects of infection. Biol Rev Camb Philos Soc. 2009;84(3):349–73. doi: 10.1111/j.1469-185X.2008.00076.x 19438430

[123] Hamilton W, Watt K. Hamilton and Watt - 1970 - Refuging.pdf.

[124] Ahrens AK, Selinka H-C, Mettenleiter TC, Beer M, Harder TC. Exploring surface water as a transmission medium of avian influenza viruses - systematic infection studies in mallards. Emerg Microbes Infect. 2022;11(1):1250–61. doi: 10.1080/22221751.2022.2065937 35473641 PMC9090351

[125] Nagy A, Černíková L, Sedlák K. Genetic data and meteorological conditions suggesting windborne transmission of H5N1 high-pathogenicity avian influenza between commercial poultry outbreaks. PLoS One. 2025;20(9):e0319880. doi: 10.1371/journal.pone.0319880 40911554 PMC12412978

[126] Bedard J, Lapointe G. Responses of hayfield vegetation to spring grazing by greater snow geese. The Journal of Applied Ecology. 1991;28(1):187. doi: 10.2307/2404124

[127] Belanger L, Bedard J. Energetic cost of man-induced disturbance to staging snow geese. The Journal of Wildlife Management. 1990;54(1):36. doi: 10.2307/3808897

[128] Nolet BA, Kölzsch A, Elderenbosch M, van Noordwijk AJ. Scaring waterfowl as a management tool: how much more do geese forage after disturbance? J Appl Ecol. 2016;53:1413–21. doi: 10.1111/1365-2664.12698

[129] Shriner SA, Root JJ. A review of avian influenza a virus associations in synanthropic birds. Viruses. 2020;12(11):1209. doi: 10.3390/v12111209 33114239 PMC7690888

[130] Caliendo V, Kleyheeg E, Beerens N, Camphuysen KCJ, Cazemier R, Elbers ARW, et al. Effect of 2020-21 and 2021-22 Highly Pathogenic Avian Influenza H5 Epidemics on Wild Birds, the Netherlands. Emerg Infect Dis. 2024;30:50–7. doi: 10.3201/eid3001.230970 38040665 PMC10756359

[131] Islam A, Islam S, Amin E, Hasan R, Hassan MM, Mia M, et al. Prevalence of avian influenza (h5 and h9) subtypes and their associated risk factors in pigeon and quail in live bird markets, Bangladesh. International Journal of Infectious Diseases. 2023;130:S71. doi: 10.1016/j.ijid.2023.04.177

[132] Moura P, Küker S, Farnell M, Stowell-Moss J, Tickel J, Buholzer P, et al. Socio-psychological and external factors influencing biosecurity compliance in U.S. poultry farming. Vet Sci. 2025;12(10):925. doi: 10.3390/vetsci12100925 41150064 PMC12567621

[133] Rolfes MA, Bauck L, Lipton BA, Margrey SF, Campagna RA, Harker E, et al. Knowledge, attitudes, and practices regarding avian influenza among owners of backyard flocks - United States, July-December 2025. MMWR Morb Mortal Wkly Rep. 2026;75(18):234–9. doi: 10.15585/mmwr.mm7518a2 42133554 PMC13175396

