# Supplementary material for: Using high frequency GPS data to assess wintering goose proximity to commercial poultry facilities on the Delmarva peninsula for avian influenza risk management and surveillance
Source: PLoS One. 2026 Sep 1;21(9):e0355415. doi: 10.1371/journal.pone.0355415 (PMC13533351; doi:10.1371/journal.pone.0355415)
Supplement: Supplementary file 1 [file pone.0355415.s006.docx]

**Response to Reviewers**

**Main Comments**

1. Please address the following additional outstanding issues as outlined by Reviewer number 3:
2. Fully and convincingly explain the implications of the imbalanced sample sizes, for the conclusions draw
3. Provide an explanation as to why the transmission pathway specificity (whether direct contact, aerosol, contamination, waterborne etc.) was not incorporated into the risk modeling procedure. If no satisfactory explanation is provided, a re-analysis process may become necessary and hence a re-write of the results, discussion, conclusion and abstract.
4. Provide a satisfactory defensible account of why you think the considerably low value of the model deviance explained (less than 30% hence low explanation power) does not impact your conclusions and render them near-speculative

**Reviewer #1**: I appreciated the opportunity to review this well-written paper that uses movement data from GPS marked geese to determine risk for poultry facilities in the Delmarva Peninsula. While the overall number of birds marked was low, the analyses used were seem to accurately predict risk for Delmarva farms and identify important predictors of goose presence at or near poultry farms. These results are important for those trying to identify what biosecurity measures/habitat management effort could benefit all farms, as well as for identifying which farms should heighten their surveillance and biosecurity measures immediately due to their risk ranking. I think this second point could be made more forcefully in the abstract. I mainly had suggestions to improve clarity but see comment on Page 11 about weighting exposure days by number of individuals. Please see specific comments below.

Reviewer Comment: Page 5: "Advances in GPS animal tracking and biologging have significantly increased capabilities of waterfowl research (Schreven et al. 2021; Askren et al. 2022; Trimbake 2023; Kölzsch et al. 2023), yet these methods have rarely been used to study waterfowl disease ecology in the U.S. (Yamaguchi et al. 2008; Takekawa et al. 2010; Cappelle et al. 2011; Lee et al. 2020; Humphreys et al. 2021; McDuie et al. 2022)." – Suggested slight rewording to improve clarity

Response to reviewer: To improve clarity, we have changed the sentence to “Advances in GPS animal tracking and biologging have greatly increased understanding of waterfowl movement, habitat use, migration, and behavioral ecology (Schreven et al. 2021; Askren et al. 2022; Trimbake 2023; Kölzsch et al. 2023). Despite these advances, relatively few studies have leveraged these technologies to address questions related to waterfowl disease ecology, particularly in the United States (Yamaguchi et al. 2008; Takekawa et al. 2010; Cappelle et al. 2011; Lee et al. 2020; Humphreys et al. 2021; McDuie et al. 2022)”

Reviewer Comment: Page 6 "The DP (Figure 2-1) is part of the Mid-Atlantic Region, encompassing eight states (New York, New Jersey, Pennsylvania, Delaware, Maryland, Virginia, West Virginia, and North Carolina) and Washington D.C." – This is a little confusing as the map in Figure 2-1 does not show all 8 states and it doesn’t appear that the DP “encompasses” 8 states. To me that would mean all 8 states are fully contained within the DP. Please provide a new map to show how much of each of the 8 states is contained in the DP or rewrite the study area description to clarify.

Response to reviewer: Removed any mention of the Mid-Atlantic because that’s misleading/confusing. I changed to: “The DP (Figure 2-1) served as the study area. Located along the Atlantic coast of the eastern United States, the DP comprises all of Delaware, Eastern Shore of Maryland, and the southern tip of Virginia.”

Reviewer Comment: Page 9: "All capture and marking procedures were approved by…." Suggested additional words to include that marking was covered by permits and ACUC too.

Response to reviewer: Added capturing and GPS attachment. All capturing and GPS attachment procedures were approved by the University of Delaware Animal Use and Care Committee …

Reviewer Comment: Page 11: "We calculated cumulative goose exposure time in “goose exposure days” by multiplying the number of points by the 5-minute interval between GPS collection and converting to days" Was this weighted at all by number of individual birds that produced the points? If not, do you think you could be overestimating exposure? Or if this is not a correct assumption, please expand on the methods and how this is not pertinent to the analysis.

Response to reviewer: Added “Goose exposure days were calculated as the cumulative time spent by all tracked geese near poultry facilities and therefore represent total goose-use time rather than exposure averaged across individuals.”

Goose exposure days were calculated as the cumulative amount of time that tracked geese spent within the specified distance of poultry facilities and were not weighted by the number of individual birds contributing observations. This was intentional because our objective was to quantify cumulative goose presence and potential exposure at facilities rather than average exposure per individual. Under this framework, time contributed by multiple birds represents additional exposure rather than pseudoreplication.

Reviewer Comment: Page 12: "To reduce spatial autocorrelation, we partitioned grid cells into 25 groups (grid cells in each group were separated by 5-km spacing)…" Wasn’t clear if grid cells within each of the 25 group were separated by 5-km or if the 25 groups themselves were separated by 5-km. Please clarify since it has implications for spatial autocorrelation.

Response to reviewer: Added “To reduce spatial autocorrelation, we partitioned grid cells into 25 groups such that grid cells within a given group were separated by at least 5 km. Modeling iterations were then conducted separately for each group, and covariate data were combined across years. We used a complete pairwise test to evaluate collinearity among predictors.”

Reviewer Comment: Page 12: For the BRT analyses I don’t see any mention of testing for model accuracy such as using the area under the curve (ex Zou et al. 2007) for resubstitution and 10-fold cross-validation methods. Was model accuracy evaluated ? Please add information here about it.

Response to reviewer: We did present the deviance explained by the models via cross-validation in the results section, but have added clarification to the Methods section describing the cross-validation procedure used to assess model performance and noting that deviance explained represents predictive performance on withheld data.. Models were iteratively trained and tested using withheld subsets of the data to optimize model complexity and minimize overfitting, and reported deviance explained values were based on cross-validated (out-of-bag) predictions rather than model fit to the training data.

Area under the curve (AUC) was not calculated because our response variable, goose exposure intensity (goose points/hour), was continuous rather than binary. AUC is primarily used to evaluate discrimination performance in classification models with binary outcomes and is therefore not applicable to the response variable analyzed in this study.

Reviewer Comment: Page 13: "One third (2,012) of all DP poultry facilities had GSGO within a buffer…." This is the first time you mention a buffer in the results and I think it would be useful to saying “the 2km buffer around each farm” instead of “a buffer” to clarify what you mean.

Response to reviewer: I have added One third (2,012) of all DP poultry facilities had GSGO “within a 2km buffer around each farm” during all winters (2019–2023). In the first mention in the results.

Reviewer Comment: Page 42 "To demonstrate the effectiveness of the percentile risk ranking system for

predicting AI outbreaks at facilities, we retrospectively assessed whether these facilities

could have been flagged prior to the H5N1 outbreak based on cumulative exposure since

the first wild bird detection in the United States. All seven facilities infected with HPAIv

in the DP were ranked within the top 25% priority farms, with one infected facility being

one of the top-25 facilities flagged based on risk (i.e., top 0.4%). The facilities classified

in the top 25% most exposed (~1,500 facilities) could be a criteria for focusing

biosecurity efforts when resources of response teams (i.e., veterinarians) are limited."

- Would be good to highlight this in the results and abstract as this is one of the best utilities of this work – identifying criteria to identify and implement preemptive biosecurity response at the most vulnerable farms

Response to reviewer: Added “All seven facilities infected with HPAIv in the DP were ranked within the top 25% priority farms, with one infected facility being one of the top-25 facilities flagged based on risk (i.e., top 0.4%).” on page 16.

Added “This could be used to guide biosecurity efforts when resources of response teams are limited.” In the Abstract

Reviewer Comment: Figures 2-4, and 2-5

These are informative panels, but labeling, etc is too small. Perhaps can drop graphs of some of the less important predictors and make the others larger?

Response to reviewer: Anything with mean relative importance <1.0 has been moved to supplementary information.

**Reviewer #2**: The manuscript “Using High Frequency GPS Data To Assess Wintering Goose Proximity To Commercial Poultry Facilities On The Delmarva Peninsula For Avian Influenza Risk Management and Surveillance” summarises the behavioural pattern of tracked wild birds over several years and use it for movement, environmental and risk mapping of avian influenza in poultry farms.

The study is thoroughly and excellently done, it will add important value and knowledge to the field. The report contains massive data which will be very useful for research and understanding animal behaviour and risk due to avian influenza.

The mayor points to consider is that it’s quite long and exhausting to read through. It might be beneficial to think about parts that could be better represented in a figure e.g. the description of the findings for the environmental and temporal variables section, to shorten the text. Also the discussion is quite long, and the main findings and points are vanishing in the magnitude of data and interpretation. Shortening and condensing to findings would be helpful for the reader to not get lost.

Reviewer Comment: The NWR abbreviation was not really introduced.

Response to reviewer: Added “hereby NWR” in first mention of NWR on page 12. We also derived distances to National Wildlife Refuges (hereby NWRs) from the USGS Protected Area Database.

Reviewer Comment: The use of such data for nowcasting and early risk assessment could be eleborated.

Response to reviewer: I have added more mention of this in the abstract and in the results section

**Reviewer #3**: This manuscript presents a four-year GPS telemetry study of Greater Snow Geese (GSGO, N=59) and Canada Geese (CANG, N=9) wintering on the Delmarva Peninsula, with the dual aim of (1) constructing a percentile-based risk ranking system for commercial poultry facilities based on waterfowl exposure, and (2) modelling the spatial, temporal, and landscape drivers of that exposure using Boosted Regression Trees (BRT). The topic is timely and of clear applied significance given the ongoing H5N1 outbreak, and the use of fine-scale GPS data (5-minute fix rate) represents a methodological strength. However, several issues require attention before the manuscript is suitable for publication.

Reviewer Comment: 1. Sample Size Imbalance and Its Implications

The most fundamental concern is the severe imbalance between the two focal species: 59 GSGO versus only 9 CANG. The authors acknowledge this briefly, but do not adequately address its consequences for the comparative claims made throughout the paper. With only 9 CANG individuals — 7 of which contributed to modelling — statements about species-level differences in habitat selection, temporal patterns, and geographic preferences carry very different levels of inferential confidence. The BRT models for CANG explained 23.79% deviance versus 16.94% for GSGO, but this difference may partly reflect overfitting given the smaller CANG sample. The authors should provide explicit uncertainty bounds on CANG-specific conclusions and temper comparative language accordingly.

Response to reviewer: We agree that the number of independent Canada Goose individuals (n = 7 used in modeling) is substantially smaller than the Greater Snow Goose sample (n = 59), and that this limits the strength of population-level inference for Canada Geese.

Added: “Mixed-effects models account for repeated observations from the same individuals through random effects. Inference is based on movement behavior while accounting for within-individual correlation.”

We found that 7.1% (429) of all DP poultry facilities had CANG detected within a buffer during all winters (2021–2023). Across all winters combined, 7 out of 9 (77.8%) GPS-marked CANG were present within at least one DP farm buffer. The smaller CAGO sample size further limits the model's species-wide representativeness relative to the larger SNGO dataset. However, geese of both species are highly social species that often move and forage in groups. Individual GPS-tagged birds may therefore provide information on the movements and habitat use of larger groups rather than isolated individuals.

We believe the comparison remains informative provided that limitations are clearly acknowledged and conclusions are appropriately qualified.

Reviewer Comment: 2. Transmission Pathway Assumptions Are Underexplored

The manuscript implicitly assumes that goose GPS proximity to a facility perimeter (within 2 km) translates meaningfully to HPAIv transmission risk. However, the actual transmission mechanism — whether through direct contact, aerosol, surface contamination, fomite transfer via workers, or waterborne routes — is not formally incorporated into the risk model. A 2-km buffer represents an epidemiologically broad zone; a goose GPS point 1,900 m from a facility perimeter conveys very different biosecurity implications than one 10 m away. The authors do note minimum distances of 1.7 m (GSGO) and 3.9 m (CANG), which is valuable, but the binary exposure classification used in the risk ranking does not reflect this gradient.

Response to reviewer: We agree that a GPS location 10 m from a facility likely represents a different level of potential interaction than one located 1,900 m away. Our objective, however, was not to estimate direct contact risk as a continuous function of distance, but rather to quantify the relative frequency with which geese occurred within a biologically relevant zone surrounding poultry facilities. We selected a 2-km threshold because it approximated the average flight distance observed among marked geese in this study (and my larger thesis dataset) and represented a distance over which an average goose could theoretically move and reach a facility in a single flight. Thus, the metric was intended to represent potential exposure opportunity rather than direct facility use. To provide additional context, we also report minimum observed distances between geese and facilities (1.7 m for Greater Snow Geese and 3.9 m for Canada Geese), which demonstrate that some birds approached facilities very closely. We have clarified this distinction in the manuscript.

I changed the text to “We counted the number of GPS locations occurring within 2 km of each facility perimeter. This threshold was selected because it approximated the average flight distance observed among marked geese in this study and represented a biologically relevant zone within which an average goose could potentially reach a facility during a single movement event. Therefore, locations within 2 km were interpreted as indicators of potential exposure opportunity rather than direct facility use.”

Added “Future research could explore risk rankings based on cumulative exposure days across multiple buffer distances and evaluate their ability to predict historical HPAI outbreaks, similar to the approach presented here using 2-km buffers.” In conclusions section.

**---------------------------------------------------------------------------------------------------------------------**

Reviewer Comment: 3. Model Deviance Explained Is Low

The BRT models explain only 16.94% (GSGO) and 23.79% (CANG) of deviance. While the authors note this in passing, the implications for the reliability of predictor importance rankings and partial response plots are not discussed. With such modest explanatory power, response curves for lower-ranked predictors (e.g., farm pond within 500 m, grassland proportion) may largely reflect noise. The authors should discuss what ecological or methodological factors might account for the unexplained variance — individual variation in movement, unmeasured habitat features, flock dynamics, hunting pressure heterogeneity — and acknowledge which conclusions are robust versus speculative given this limitation.

Response to reviewer:

Recalculation changed the mean cross-validated deviance explained to 16.68 ± 0.5% and 25.17 ± 2.65% for GSGO and CANG respectively.

We acknowledge that the BRT models explained a modest proportion of deviance (16.94% for GSGO and 23.79% for CANG). However, these values represent deviance explained on cross-validated (out-of-bag) data rather than the training dataset itself. We used cross-validation during model fitting to iteratively optimize model complexity and minimize overfitting, thereby emphasizing predictive performance on independent data rather than maximizing fit to the training observations.

Added “We could have used an alternative framework involving mixed-effects models that account for repeated observations from the same individuals through random effects. In such models, inference is based on movement behavior while accounting for within-individual correlation. Instead, we fit models using unaggregated, individual-level observations to maintain data granularity and preclude any ecological fallacy resulting from cross-level inference [63]. Although hierarchical or temporal data aggregation typically yields higher goodness-of-fit metrics by stripping out within-group variance [64], it masks critical individual stochasticity and unobserved behavioral heterogeneity [65,66]. By retaining the full scope of variation within and across individuals, we can more precisely assess why an individual goose is near a farm, without the potential distortion from population-level aggregation. Thus, the lower deviance explained reported herein is a structural mathematical consequence of our modeling choice to retain disaggregated variance components, rather than a failure of the model to capture meaningful underlying patterns. This approach ensures unbiased parameter estimates and maintains the ecological validity of the inference." To methods section.

Added: “Although the BRT models explained a modest proportion of deviance, this level of explanatory power is not uncommon for telemetry-based studies of animal movement, where behavior is influenced by numerous factors operating across spatial and temporal scales. Telemetry datasets inherently contain substantial variability among individuals and flocks, and much of this variation cannot be captured using landscape-scale environmental covariates alone. Unexplained variance may reflect factors such as individual behavioral differences, daily flock dynamics, temporary food availability, crop type and harvest status, waste grain abundance, hunting pressure, disturbance, fine-scale weather conditions, small water features not represented in national datasets, land management activities, and roost-site fidelity. Despite this unexplained variation, the models consistently identified a small set of dominant predictors across cross-validation iterations, providing confidence in the ecological relevance of the highest-ranked variables (relative importance >1.0). Consequently, our conclusions are focused primarily on these strong and consistent predictors of winter habitat use, whereas interpretations of lower-ranked predictors should be viewed more cautiously due to their comparatively weak influence and greater sensitivity to unmeasured sources of variation. Citations provided for the above,
