# Supplementary material for: Using high frequency GPS data to assess wintering goose proximity to commercial poultry facilities on the Delmarva peninsula for avian influenza risk management and surveillance
Source: PLoS One. 2026 Sep 1;21(9):e0355415. doi: 10.1371/journal.pone.0355415 (PMC13533351; doi:10.1371/journal.pone.0355415)
Supplement: Peer review history

# Peer review: Decision Letter 0

**DOI:** 10.1371/journal.pone.0355415.r001

**Authors:** Nickson Erick Otieno

22 May 2026

Dear Dr. Hardy,

Thank you for submitting your manuscript to PLOS ONE. After careful consideration, we feel that it has merit but does not fully meet PLOS ONE’s publication criteria as it currently stands. Therefore, we invite you to submit a revised version of the manuscript that addresses the points raised during the review process.

==============================

Thank you for addressing the initial review concerns.

Please address the following additional outstanding issues as outlined by Reviewer number 3:

Fully and convincingly explain the implications of the imbalanced sample sizes, for the conclusions drawn

Provide an explanation as to why the transmission pathway specificity (whether direct contact, aerosol, contamination, waterborne etc.) was not incorporated into the risk modeling procedure. If no satisfactory explanation is provided, a re-analysis process may become necessary and hence a re-write of the results, discussion, conclusion and abstract.

Provide a satisfactory defensible account of why you think the considerably low value of the model deviance explained (less than 30% hence low explanation power) does not impact your conclusions and render them near-speculative

==============================

Please submit your revised manuscript by Jul 06 2026 11:59PM. If you will need more time than this to complete your revisions, please reply to this message or contact the journal office at plosone@plos.org. When you're ready to submit your revision, log on to https://www.editorialmanager.com/pone/ and select the 'Submissions Needing Revision' folder to locate your manuscript file.

- A letter that responds to each point raised by the academic editor and reviewer(s). You should upload this letter as a separate file labeled 'Response to Reviewers'.
- A marked-up copy of your manuscript that highlights changes made to the original version. You should upload this as a separate file labeled 'Revised Manuscript with Track Changes'.
- An unmarked version of your revised paper without tracked changes. You should upload this as a separate file labeled 'Manuscript'.

If you would like to make changes to your financial disclosure, please include your updated statement in your cover letter. Guidelines for resubmitting your figure files are available below the reviewer comments at the end of this letter.

If applicable, we recommend that you deposit your laboratory protocols in protocols.io to enhance the reproducibility of your results. Protocols.io assigns your protocol its own identifier (DOI) so that it can be cited independently in the future. For instructions see: https://journals.plos.org/plosone/s/submission-guidelines#loc-laboratory-protocols. Additionally, PLOS ONE offers an option for publishing peer-reviewed Lab Protocol articles, which describe protocols hosted on protocols.io. Read more information on sharing protocols at https://plos.org/protocols?utm_medium=editorial-email&utm_source=authorletters&utm_campaign=protocols.

As the corresponding author, your ORCID iD is verified in the submission system and will appear in the published article. PLOS supports the use of ORCID, and we encourage all coauthors to register for an ORCID iD and use it as well. Please encourage your coauthors to verify their ORCID iD within the submission system before final acceptance, as unverified ORCID iDs will not appear in the published article. *Only*  the individual author can complete the verification step; PLOS staff *cannot*  verify ORCID iDs on behalf of authors.

We look forward to receiving your revised manuscript.

Kind regards,

Nickson Erick Otieno, PhD

Academic Editor

PLOS One

Journal Requirements:

When submitting your revision, we need you to address these additional requirements.

1. Please ensure that your manuscript meets PLOS ONE's style requirements, including those for file naming. The PLOS ONE style templates can be found at

https://journals.plos.org/plosone/s/file?id=wjVg/PLOSOne_formatting_sample_main_body.pdf and https://journals.plos.org/plosone/s/file?id=ba62/PLOSOne_formatting_sample_title_authors_affiliations.pdf

2. Please note that PLOS One has specific guidelines on code sharing for submissions in which author-generated code underpins the findings in the manuscript. In these cases, we expect all author-generated code to be made available without restrictions upon publication of the work. Please review our guidelines at https://journals.plos.org/plosone/s/materials-and-software-sharing#loc-sharing-code and ensure that your code is shared in a way that follows best practice and facilitates reproducibility and reuse.

3. Please provide a complete Data Availability Statement in the submission form, ensuring you include all necessary access information or a reason for why you are unable to make your data freely accessible. If your research concerns only data provided within your submission, please write "All data are in the manuscript and/or supporting information files" as your Data Availability Statement.

4. When completing the data availability statement of the submission form, you indicated that you will make your data available on acceptance. We strongly recommend all authors decide on a data sharing plan before acceptance, as the process can be lengthy and hold up publication timelines. Please note that, though access restrictions are acceptable now, your entire data will need to be made freely accessible if your manuscript is accepted for publication. This policy applies to all data except where public deposition would breach compliance with the protocol approved by your research ethics board. If you are unable to adhere to our open data policy, please kindly revise your statement to explain your reasoning and we will seek the editor's input on an exemption. Please be assured that, once you have provided your new statement, the assessment of your exemption will not hold up the peer review process.

5. We note that your Data Availability Statement is currently as follows: Goose GPS tracking data is stored on a privately owned Movebank account with ID - 1509697502

All other relevant data are within the manuscript and its Supporting Information files.

Please confirm at this time whether or not your submission contains all raw data required to replicate the results of your study. Authors must share the “minimal data set” for their submission. PLOS defines the minimal data set to consist of the data required to replicate all study findings reported in the article, as well as related metadata and methods (https://journals.plos.org/plosone/s/data-availability#loc-minimal-data-set-definition).

For example, authors should submit the following data:

- The values behind the means, standard deviations and other measures reported;

- The values used to build graphs;

- The points extracted from images for analysis.

Authors do not need to submit their entire data set if only a portion of the data was used in the reported study.

If your submission does not contain these data, please either upload them as Supporting Information files or deposit them to a stable, public repository and provide us with the relevant URLs, DOIs, or accession numbers. For a list of recommended repositories, please see https://journals.plos.org/plosone/s/recommended-repositories.

If there are ethical or legal restrictions on sharing a de-identified data set, please explain them in detail (e.g., data contain potentially sensitive information, data are owned by a third-party organization, etc.) and who has imposed them (e.g., an ethics committee). Please also provide contact information for a data access committee, ethics committee, or other institutional body to which data requests may be sent. If data are owned by a third party, please indicate how others may request data access.

6. We note that the grant information you provided in the ‘Funding Information’ and ‘Financial Disclosure’ sections do not match.

When you resubmit, please ensure that you provide the correct grant numbers for the awards you received for your study in the ‘Funding Information’ section.

7. Thank you for stating the following financial disclosure:

Funding – We would like to thank the U.S. Department of Agriculture —National Institute of Food and Agriculture for project funding (grant no. 2020-68014-30975 and A21-0253-S002).

The Natural Sciences and Engineering Council of Canada (RGPIN- 2019-05185)

The Fonds de recherche du Quebec Nature et Technologies (271544)

Sentinelle Nord Program from the Canada First Research Excellence Fund and the Network of Centre of Excellence of Canada (ArcticNET; PP12)

Please state what role the funders took in the study. If the funders had no role, please state: "The funders had no role in study design, data collection and analysis, decision to publish, or preparation of the manuscript."

If this statement is not correct you must amend it as needed.

Please include this amended Role of Funder statement in your cover letter; we will change the online submission form on your behalf.

8. We note that Figure(s) 2-1, 2-2, 2-3, Appendix B in your submission contain [map/satellite] images which may be copyrighted. All PLOS content is published under the Creative Commons Attribution License (CC BY 4.0), which means that the manuscript, images, and Supporting Information files will be freely available online, and any third party is permitted to access, download, copy, distribute, and use these materials in any way, even commercially, with proper attribution. For these reasons, we cannot publish previously copyrighted maps or satellite images created using proprietary data, such as Google software (Google Maps, Street View, and Earth). For more information, see our copyright guidelines: http://journals.plos.org/plosone/s/licenses-and-copyright.

We require you to either (1) present written permission from the copyright holder to publish these figures specifically under the CC BY 4.0 license, or (2) remove the figures from your submission:

a. You may seek permission from the original copyright holder of Figure(s) 2-1, 2-2, 2-3, Appendix B to publish the content specifically under the CC BY 4.0 license.

We recommend that you contact the original copyright holder with the Content Permission Form (http://journals.plos.org/plosone/s/file?id=7c09/content-permission-form.pdf) and the following text:

“I request permission for the open-access journal PLOS ONE to publish XXX under the Creative Commons Attribution License (CCAL) CC BY 4.0 (http://creativecommons.org/licenses/by/4.0/). Please be aware that this license allows unrestricted use and distribution, even commercially, by third parties. Please reply and provide explicit written permission to publish XXX under a CC BY license and complete the attached form.”

Please upload the completed Content Permission Form or other proof of granted permissions as an "Other" file with your submission.

In the figure caption of the copyrighted figure, please include the following text: “Reprinted from [ref] under a CC BY license, with permission from [name of publisher], original copyright [original copyright year].”

b. If you are unable to obtain permission from the original copyright holder to publish these figures under the CC BY 4.0 license or if the copyright holder’s requirements are incompatible with the CC BY 4.0 license, please either i) remove the figure or ii) supply a replacement figure that complies with the CC BY 4.0 license. Please check copyright information on all replacement figures and update the figure caption with source information. If applicable, please specify in the figure caption text when a figure is similar but not identical to the original image and is therefore for illustrative purposes only.

The following resources for replacing copyrighted map figures may be helpful:

USGS National Map Viewer (public domain): http://viewer.nationalmap.gov/viewer/

The Gateway to Astronaut Photography of Earth (public domain): http://eol.jsc.nasa.gov/sseop/clickmap/

Maps at the CIA (public domain): https://www.cia.gov/library/publications/the-world-factbook/index.html and https://www.cia.gov/library/publications/cia-maps-publications/index.html

NASA Earth Observatory (public domain): http://earthobservatory.nasa.gov/

Landsat: http://landsat.visibleearth.nasa.gov/

USGS EROS (Earth Resources Observatory and Science (EROS) Center) (public domain): http://eros.usgs.gov/#

Natural Earth (public domain): http://www.naturalearthdata.com/

9. We notice that your supplementary [figures/tables] are included in the manuscript file. Please remove them and upload them with the file type 'Supporting Information'. Please ensure that each Supporting Information file has a legend listed in the manuscript after the references list.

10. If the reviewer comments include a recommendation to cite specific previously published works, please review and evaluate these publications to determine whether they are relevant and should be cited. There is no requirement to cite these works unless the editor has indicated otherwise.

11. Please review your reference list to ensure that it is complete and correct. If you have cited papers that have been retracted, please include the rationale for doing so in the manuscript text, or remove these references and replace them with relevant current references. Any changes to the reference list should be mentioned in the rebuttal letter that accompanies your revised manuscript. If you need to cite a retracted article, indicate the article’s retracted status in the References list and also include a citation and full reference for the retraction notice.

[Note: HTML markup is below. Please do not edit.]

Reviewers' comments:

Reviewer's Responses to Questions

**Comments to the Author**

1. Is the manuscript technically sound, and do the data support the conclusions?

Reviewer #1: Yes

Reviewer #2: Yes

Reviewer #3: Yes

**********

2. Has the statistical analysis been performed appropriately and rigorously?

Reviewer #1: Yes

Reviewer #2: Yes

Reviewer #3: Yes

**********

3. Have the authors made all data underlying the findings in their manuscript fully available?

The PLOS Data policy

Reviewer #1: Yes

Reviewer #2: Yes

Reviewer #3: Yes

**********

4. Is the manuscript presented in an intelligible fashion and written in standard English?

Reviewer #1: Yes

Reviewer #2: Yes

Reviewer #3: Yes

**********

Reviewer #1: I appreciated the opportunity to review this well-written paper that uses movement data from GPS marked geese to determine risk for poultry facilities in the Delmarva Peninsula. While the overall number of birds marked was low, the analyses used were seem to accurately predict risk for Delmarva farms and identify important predictors of goose presence at or near poultry farms. These results are important for those trying to identify what biosecurity measures/habitat management effort could benefit all farms, as well as for identifying which farms should heighten their surveillance and biosecurity measures immediately due to their risk ranking. I think this second point could be made more forcefully in the abstract. I mainly had suggestions to improve clarity but see comment on Page 11 about weighting exposure days by number of individuals. Please see specific comments below.

Page 5: "Advances in GPS animal tracking and biologging have significantly increased

capabilities of waterfowl research (Schreven et al. 2021; Askren et al. 2022; Trimbake

2023; Kölzsch et al. 2023), yet these methods have rarely been used to study waterfowl disease ecology in the U.S. (Yamaguchi et al. 2008; Takekawa et al. 2010; Cappelle et al. 2011; Lee et al. 2020; Humphreys et al. 2021; McDuie et al. 2022)." – Suggested slight rewording to improve clarity

Page 6 "The DP (Figure 2-1) is part of the Mid-Atlantic Region, encompassing eight states (New York, New Jersey, Pennsylvania, Delaware, Maryland, Virginia, West Virginia, and

North Carolina) and Washington D.C." – This is a little confusing as the map in Figure 2-1 does not show all 8 states and it doesn’t appear that the DP “encompasses” 8 states. To me that would mean all 8 states are fully contained within the DP. Please provide a new map to show how much of each of the 8 states is contained in the DP or rewrite the study area description to clarify.

Page 9: "All capture and marking procedures were approved by…." Suggested additional words to include that marking was covered by permits and ACUC too.

Page 11: "We calculated cumulative goose exposure time in “goose exposure days” by multiplying the number of points by the 5-minute interval between GPS collection and converting to days" Was this weighted at all by number of individual birds that produced the points? If not, do you think you could be overestimating exposure? Or if this is not a correct assumption, please expand on the methods and how this is not pertinent to the analysis.

Page 12: "To reduce spatial autocorrelation, we partitioned grid cells into 25 groups (grid cells in each group were separated by 5-km spacing)…" Wasn’t clear if grid cells within each of the 25 group were separated by 5-km or if the 25 groups themselves were separated by 5-km. Please clarify since it has implications for spatial autocorrelation.

Page 12: For the BRT analyses I don’t see any mention of testing for model accuracy such as using the area under the curve (ex Zou et al. 2007) for resubstitution and 10-fold cross-validation methods. Was model accuracy evaluated ? Please add information here about it.

Page 13: "One third (2,012) of all DP poultry facilities had GSGO within a buffer…." This is the first time you mention a buffer in the results and I think it would be useful to saying “the 2km buffer around each farm” instead of “a buffer” to clarify what you mean.

Page 42 "To demonstrate the effectiveness of the percentile risk ranking system for

predicting AI outbreaks at facilities, we retrospectively assessed whether these facilities

could have been flagged prior to the H5N1 outbreak based on cumulative exposure since

the first wild bird detection in the United States. All seven facilities infected with HPAIv

in the DP were ranked within the top 25% priority farms, with one infected facility being

one of the top-25 facilities flagged based on risk (i.e., top 0.4%). The facilities classified

in the top 25% most exposed (~1,500 facilities) could be a criteria for focusing

biosecurity efforts when resources of response teams (i.e., veterinarians) are limited."

- Would be good to highlight this in the results and abstract as this is one of the best utilities of this work – identifying criteria to identify and implement preemptive biosecurity response at the most vulnerable farms

Figures 2-4, and 2-5

These are informative panels, but labeling, etc is too small. Perhaps can drop graphs of some of the less important predictors and make the others larger?

Reviewer #2: The manuscript “Using High Frequency GPS Data To Assess Wintering Goose Proximity To Commercial Poultry Facilities On The Delmarva Peninsula For Avian Influenza Risk Management and Surveillance” summarises the behavioural pattern of tracked wild birds over several years and use it for movement, environmental and risk mapping of avian influenza in poultry farms.

The study is thoroughly and excellently done, it will add important value and knowledge to the field. The report contains massive data which will be very useful for research and understanding animal behaviour and risk due to avian influenza.

The mayor points to consider is that it’s quite long and exhausting to read through. It might be beneficial to think about parts that could be better represented in a figure e.g. the description of the findings for the environmental and temporal variables section, to shorten the text. Also the discussion is quite long, and the main findings and points are vanishing in the magnitude of data and interpretation. Shortening and condensing to findings would be helpful for the reader to not get lost.

The NWR abbreviation was not really introduced.

The use of such data for nowcasting and early risk assessment could be eleborated.

Reviewer #3: This manuscript presents a four-year GPS telemetry study of Greater Snow Geese (GSGO, N=59) and Canada Geese (CANG, N=9) wintering on the Delmarva Peninsula, with the dual aim of (1) constructing a percentile-based risk ranking system for commercial poultry facilities based on waterfowl exposure, and (2) modelling the spatial, temporal, and landscape drivers of that exposure using Boosted Regression Trees (BRT). The topic is timely and of clear applied significance given the ongoing H5N1 outbreak, and the use of fine-scale GPS data (5-minute fix rate) represents a methodological strength. However, several issues require attention before the manuscript is suitable for publication.

1. Sample Size Imbalance and Its Implications

The most fundamental concern is the severe imbalance between the two focal species: 59 GSGO versus only 9 CANG. The authors acknowledge this briefly, but do not adequately address its consequences for the comparative claims made throughout the paper. With only 9 CANG individuals — 7 of which contributed to modelling — statements about species-level differences in habitat selection, temporal patterns, and geographic preferences carry very different levels of inferential confidence. The BRT models for CANG explained 23.79% deviance versus 16.94% for GSGO, but this difference may partly reflect overfitting given the smaller CANG sample. The authors should provide explicit uncertainty bounds on CANG-specific conclusions and temper comparative language accordingly.

2. Transmission Pathway Assumptions Are Underexplored

The manuscript implicitly assumes that goose GPS proximity to a facility perimeter (within 2 km) translates meaningfully to HPAIv transmission risk. However, the actual transmission mechanism — whether through direct contact, aerosol, surface contamination, fomite transfer via workers, or waterborne routes — is not formally incorporated into the risk model. A 2-km buffer represents an epidemiologically broad zone; a goose GPS point 1,900 m from a facility perimeter conveys very different biosecurity implications than one 10 m away. The authors do note minimum distances of 1.7 m (GSGO) and 3.9 m (CANG), which is valuable, but the binary exposure classification used in the risk ranking does not reflect this gradient.

3. Model Deviance Explained Is Low

The BRT models explain only 16.94% (GSGO) and 23.79% (CANG) of deviance. While the authors note this in passing, the implications for the reliability of predictor importance rankings and partial response plots are not discussed. With such modest explanatory power, response curves for lower-ranked predictors (e.g., farm pond within 500 m, grassland proportion) may largely reflect noise. The authors should discuss what ecological or methodological factors might account for the unexplained variance — individual variation in movement, unmeasured habitat features, flock dynamics, hunting pressure heterogeneity — and acknowledge which conclusions are robust versus speculative given this limitation.

**********

what does this mean?). If published, this will include your full peer review and any attached files.

If you choose “no”, your identity will remain anonymous but your review may still be made public.

**Do you want your identity to be public for this peer review?**  For information about this choice, including consent withdrawal, please see our Privacy Policy

Reviewer #1: No

Reviewer #2: No

Reviewer #3: No

**********

[NOTE: If reviewer comments were submitted as an attachment file, they will be attached to this email and accessible via the submission site. Please log into your account, locate the manuscript record, and check for the action link "View Attachments". If this link does not appear, there are no attachment files.]

To ensure your figures meet our technical requirements, please review our figure guidelines: https://journals.plos.org/plosone/s/figures

You may also use PLOS’s free figure tool, NAAS, to help you prepare publication quality figures: https://journals.plos.org/plosone/s/figures#loc-tools-for-figure-preparation.

NAAS will assess whether your figures meet our technical requirements by comparing each figure against our figure specifications.

# Author comment: Author response to Decision Letter 1

**DOI:** 10.1371/journal.pone.0355415.r002

20 Jul 2026

Please refer to the Response_to_Reviewers_7_17_2026 document.

Attachment Submitted filename: Response_to_Reviewers_7_17_2026.docx

# Editor report: Decision Letter 1

**DOI:** 10.1371/journal.pone.0355415.r003

**Authors:** Nickson Erick Otieno

21 Jul 2026

Using High Frequency GPS Data To Assess Wintering Goose Proximity To Commercial Poultry Facilities On The Delmarva Peninsula For Avian Influenza Risk Management and Surveillance

PONE-D-26-14656R1

Dear Dr. Hardy,

We’re pleased to inform you that your manuscript has been judged scientifically suitable for publication and will be formally accepted for publication once it meets all outstanding technical requirements.

Within one week, you’ll receive an e-mail detailing the required amendments. When these have been addressed, you’ll receive a formal acceptance letter and your manuscript will be scheduled for publication.

An invoice will be generated when your article is formally accepted. Please note, if your institution has a publishing partnership with PLOS and your article meets the relevant criteria, all or part of your publication costs will be covered. Please make sure your user information is up-to-date by logging into Editorial Manager at Editorial Manager® and clicking the ‘Update My Information' link at the top of the page. For questions related to billing, please contact billing support.

If your institution or institutions have a press office, please notify them about your upcoming paper to help maximize its impact. If they’ll be preparing press materials, please inform our press team as soon as possible -- no later than 48 hours after receiving the formal acceptance. Your manuscript will remain under strict press embargo until 2 pm Eastern Time on the date of publication. For more information, please contact onepress@plos.org.

Kind regards,

Nickson Erick Otieno, PhD

Academic Editor

PLOS One

Additional Editor Comments (optional):

To finally prepare the article quality towards readiness for publication in the PLOS One, the authors need to resolve the following very few additional  minor issues

Format the cover page strictly according to the journal’s submission guidelines

Move the material describing Study Area from Introduc65ion to Methods

Remove all figures from manuscript body and submit as separate high-resolution (350-600 dpi) tiff files, leaving on figure captions in the positions where the figures currently area

Format captions for all figures and all supporting information material captions according to journal guidelines

The authors’ response to the reviewer concern as to why infection pathways were not included in modeling, are provided in the Response letter but the relevant section/part in the manuscript body itself has not been updated accordingly in a clear, explicit and defensible way

In most of the figures in main document and in supporting material, the text in the plots as well as the axis labels are not clearly legible and must be increased

All figure captions must be at the bottom and table legends at the top positions, respectively

Reviewers' comments:

# Editor report: Acceptance letter

**DOI:** 10.1371/journal.pone.0355415.r004

**Authors:** Nickson Erick Otieno

PONE-D-26-14656R1

PLOS One

Dear Dr. Hardy,

I'm pleased to inform you that your manuscript has been deemed suitable for publication in PLOS One. Congratulations! Your manuscript is now being handed over to our production team.

At this stage, our production department will prepare your paper for publication. This includes ensuring the following:

* All references, tables, and figures are properly cited

* All relevant supporting information is included in the manuscript submission,

* There are no issues that prevent the paper from being properly typeset

You will receive further instructions from the production team, including instructions on how to review your proof when it is ready. Please keep in mind that we are working through a large volume of accepted articles, so please give us a few days to review your paper and let you know the next and final steps.

Lastly, if your institution or institutions have a press office, please let them know about your upcoming paper now to help maximize its impact. If they'll be preparing press materials, please inform our press team within the next 48 hours. Your manuscript will remain under strict press embargo until 2 pm Eastern Time on the date of publication. For more information, please contact onepress@plos.org.

You will receive an invoice from PLOS for your publication fee after your manuscript has reached the completed accept phase. If you receive an email requesting payment before acceptance or for any other service, this may be a phishing scheme. Learn how to identify phishing emails and protect your accounts at https://explore.plos.org/phishing.

If we can help with anything else, please email us at customercare@plos.org.

Thank you for submitting your work to PLOS One and supporting open access.

Kind regards,

PLOS One Editorial Office Staff

on behalf of

Dr. Nickson Erick Otieno

Academic Editor

PLOS One
